# Molecular docking to homology models of human and *Trypanosoma brucei* ERK8 that identified ortholog-specific inhibitors

**DOI:** 10.1371/journal.pntd.0013487

**Published:** 2025-09-12

**Authors:** Matthew Merski, Zachary B. Mackey

**Affiliations:** 1 i3S - Instituto de Investigação e Inovação em Saúde, Universidade do Porto, Porto, Portugal; 2 IBMC – Instituto de Biologia Molecular Celular, Universidade do Porto, Porto, Portugal; 3 Institute for Evolution and Biodiversity, University of Münster, Münster, Germany; 4 Department of Biochemistry, Virginia Tech, Blacksburg, Virginia, United States of America; 5 Virginia Tech Center for Drug Discovery, Virginia Tech, Blacksburg, Virginia, United States of America; University of Sao Paulo: Universidade de Sao Paulo, BRAZIL

## Abstract

Human African Trypanosomiasis (HAT), also known as sleeping sickness, is a lethal disease caused by two vector-borne parasites: *Trypanosoma brucei gambiense* and *Trypanosoma brucei rhodesiense*. The limited number of antitrypanosomal therapies for treating these deadly parasites suffer from toxicity, poor efficacy, and unspecified targets; thus, more and better medicines are needed. We used *in silico* methods to predict features of the bioactive compound AZ960 that make it an ortholog-specific inhibitor for the extracellular-signal regulated kinase 8 of *T. brucei* (TbERK8). Our homology models showed that the TbERK8 ATP binding pocket was smaller and more hydrophobic than that of human ERK8 (HsERK8). Molecular docking studies predicted six FDA-approved compounds that would be orthologue-specific inhibitors of HsERK8 or TbERK8. Experimental testing of these compounds identified prednisolone as an HsERK8-specific inhibitor. Sildenafil inhibited TbERK8, as predicted by our binding model. Its impact on TbERK8 activity supports our hypothesis that designing compounds that can exploit differences in the orthologs as buildable scaffolds and expand the repertoire of ortholog-specific antitrypanosomal agents.

## Author summary

The discovery and production of safe and effective therapeutics for treating people suffering from neglected tropical diseases, such as sleeping sickness caused by the pathogen *Trypanosoma brucei*, is infrequently pursued. The risk of a loss or poor return on investment for the expensive drug discovery and development process for such diseases is high. Lower cost *in silico* strategies, such as molecular docking to homology models of protein targets, are an economical option for discovering lead molecules that can be developed into safe and effective therapeutics. This study describes our approach of combining molecular docking and homology modeling of extracellular-signal regulated kinases 8 (ERK8s) from *T. brucei* and human cells to discover compounds that selectively inhibit these orthologs. Experimental testing demonstrated that this approach identified FDA-approved drugs that selectively inhibited human ERK8. Our *in silico* approach predicted lead molecules that can selectively inhibit the *T. brucei* ERK8 ortholog, exploiting charge and volume differences in its binding pocket.

## Introduction

Two vector-borne parasites, *Trypanosoma brucei gambiense* and *T. b. rhodesiense,* are the causative agents of the neglected tropical disease Human African Trypanosomiasis (HAT), also known as sleeping sickness. These parasites, transmitted by the bites of infected tsetse flies, cause a lethal meningoencephalitis disease and threaten approximately 65 million people in sub-Saharan Africa. However, *T. b. gambiense* accounts for 95–97% of the reported cases [1].

The majority of antitrypanosomal therapeutics have non-specified targets, are difficult to administer, suffer from toxic side effects, and have poor efficacy. Melarsoprol is the only antitrypanosomal effective against *T. b. gambiense* and *T. b. rhodesiense* infections after they invade the brain [2]. The effectiveness of three other approved therapeutics (*e.g.,* eflornithine, suramin, and pentamidine) depends on the disease stage and the trypanosome species [3]. Eflornithine is the only antitrypanosomal with a defined molecular target (ornithine decarboxylase) and known mechanism of action [4,5].

FDA approval of the oral antitrypanosomal drug fexinidazole represents a significant advancement in treating *T. b. gambiense* [5]. However, fexinidazole is ineffective against severe brain infections or *T. b. rhodesiense* infections [1,6,7]. Patients treated with fexinidazole suffer from relapse parasitemia and the possibility of later drug resistance [6]. The limited options for antitrypanosomal therapeutics highlight an urgent need for more safe and effective HAT drugs.

One strategy for discovering new therapeutics is inhibiting known molecular targets essential for the parasite (*e.g.,* kinases that regulate essential cellular processes such as cell cycle control [8]). While targeting the kinases can be difficult, recent studies have focused on the ATP-binding site, indicating that other potential drug design strategies might also have high target specificity [9–13]. Targeting *T. brucei* kinases could be a cost-effective approach to drug discovery [14]. Given the role of mitogen-activated protein kinases (MAPKs) in response to essential extracellular signaling processes such as mitogenic signals, pro-inflammatory cytokines, and cellular stress in eukaryotes, their members hold potential for therapeutic exploitation [15].

The extracellular-signal regulated kinase 8 (ERK8) is the most recently identified member of the MAPK family [16]. The ERK8 kinase family members are unique because they do not require an upstream activating kinase. They are activated in the cell by autophosphorylation at two residues in the conserved TXY motif within its activation loop [16–19]. The product of the *T. brucei* gene Tb927.10.5140 was identified from an RNA interference screen (RNAi) to identify kinases critical for this parasite to proliferate normally [17]. This gene product, also known as MAPK6 [18], is referred to here as TbERK8 because it phylogenetically clusters into the same clade as human ERK8 (HsERK8) [19]. Knockdown of TbERK8 mRNA expression in *T. brucei* or inhibition of its activity by the ortholog-specific compound AZ960 killed the parasite [17,20], indicating TbERK8’s potential as a therapeutic target. The compound AZ960 contains methyl-pyrazole and fluorophenyl moieties [20] and is a potent, specific inhibitor of TbERK8 with *T. brucei* bioactivity, inhibiting HsERK8 poorly [20]. Conversely, Ro318220, which contains pyrrole-dione and methyl-sulfenyl moieties, shows 100–300-fold greater potency inhibiting HsERK8 than TbERK8 [19]. The selectivity of these small molecules for TbERK8 or HsERK8 indicates that they exploit divergent structural and biochemical factors in the kinase domain of these ERK8 orthologs from two evolutionarily divergent species. Therefore, understanding the structural and biochemical differences between TbERK8 and HsERK8 is necessary to predict the properties of ortholog-specific inhibitors.

*In silico* techniques allow for theoretical analyses into protein-ligand binding and are critical for drug discovery. They accelerate drug design by supporting and enriching *in vivo* and *in vitro* strategies [21,22]. Detailed, atomistic characterization of the ATP-binding pockets of ERK8 orthologs by *in silico* techniques can predict specific chemical characteristics of their ATP-binding pockets necessary for discovering or developing orthologue-selective inhibitor designs [23]. The homology model of HsERK8 by Strambi et al. [24] showed that computational techniques analyzing structural features represent a viable approach for discovering ATP-competitive inhibitors. Neither HsERK8 nor TbERK8 has an experimentally resolved structure, indicating the need for *in silico* approaches to ascertain a more detailed understanding of how small molecules selectively inhibit their activity, aided by experiments. We applied homology modeling, molecular docking, and free energy calculations of ligand-protein complexes to TbERK8 and HsERK8. These methodologies predicted the FDA-approved drugs idarubicin, fludrocortisone, prednisolone, and sildenafil as the top agents that would exploit differences between the ATP-binding pocket of TbERK8 and HsERK8. We screened these compounds against both enzymes and found differences that will allow a buildable scaffold based on AZ960 for orthologue-specific compounds that could be used to expand the repertoire of compounds for studying TbERK8 and developing safe and effective *T. brucei* therapeutics.

## Materials and methods

### Homology modeling of TbERK8 and HsERK8

Sequences for TbERK8 (Tb927.10.5140) and HsERK8 were retrieved from GeneDB [25] and GenBank [26], respectively. We used only the kinase domains, TbERK8 (residues 6–341) and HsERK8 (residues 12–345), as determined by Valenciano et al. [19] and Strambi et al. [24], to generate homology models for this study. The X-ray diffraction-resolved structure of MAPK Fus3 from *Saccharomyces cerevisiae* (PDB ID: 2b9f) [27] was included in this work due to its similarity to the ERK8 orthologues and because it was co-crystallized with ADP. The Fus3 structure was energy-minimized and validated in the same way as the homology models. We also used the crystal structure of MAPK from *Cryptosporidium parvum* (PDB ID: 3oz6) as a template. Models of TbERK8 and HsERK8 were energy-minimized using MOE [28] with the AmberEHT force field [29]. Model quality was validated with Verify3D using a variety of metrics to assess side-chain positioning [30], local environment favorability with ANOLEA [31,32], similarity to known experimentally solved structures (ProSA) [33], and backbone and torsional energies (SWISS-MODEL [34–36] (S1-S3 Fig).

### Physicochemical characterization of the ATP-binding site

The TbERK8 and HsERK8 energy-minimized homology models were evaluated to determine their interaction abilities, pocket volume, and hydrophobicity. MetaPocket 2.0 [37] was used to predict the locations of binding cavities and provide coordinates of the space-filling regions of these cavities. Coordinates of the binding site were then imported into UCSF Chimera [38] for volume calculations. Putative binding cavity spheres were manually evaluated, and regions outside the ATP-binding cavity were eliminated. The overall surface of the binding pocket was evaluated for hydrophilicity, hydrophobicity, hydrogen bonding abilities, charged areas, and pharmacophore hypothesis testing using Schrödinger-Maestro [39] to predict the receptor features required for ATP binding. Each model was analyzed using the receptor-based method to identify the ideal characteristics for orthologue-specific small molecules. Protein electrostatic surfaces were created using PyMOL [39]. Electrostatic surfaces of inhibitors and ligands used in molecular docking were created using UCSF Chimera [38] by displaying the protein surface using the Coulombic surface coloring feature. Small-molecule rotatable bond analysis was performed using AutoDock Tools [40].

### Molecular docking

We used AutoDock Tools [40] to prepare the receptor (kinase) and ligand files for docking experiments. AutoDock Vina [41] was used to dock all ligands and inhibitors used in this work, including ADP and ATP, into the energy-minimized Fus3 structure and ERK8 homology models. The ERK8 models were overlaid on Fus3 before docking to regularize grid box size and center position, given the similarities in their ADP/ATP binding cavities to Fus3. The grid box was centered on the ADP/ATP binding site based on the locations and proximity of key residues interacting with ADP in the Fus3 structure. We used a larger (22 Å x 22 Å x 22 Å) grid box in the docking process to account for differences in size between the ADP/ATP binding cavities of the three proteins. Root-mean-square deviation (RMSD) was calculated using an in-house script to evaluate the accuracy of the re-docking. Structures of AZ960 ((S)-5-Fluoro-2-(1-(4-fluorophenyl)ethylamino)-6-(5-methyl-1H-pyrazol-3-ylamino)nicotinonitrile) and Ro318220 (3-{3-[4-(1-Methyl-1*H*-indol-3-yl)-2,5-dioxo-2,5-dihydro-1*H*-pyrrol-3-yl]-1*H*-indol-1-yl}propyl carbamimidothioate), were downloaded from PubChem [42] and also docked into TbERK8 and HsERK8. Each docking experiment resulted in nine poses. The lowest energy poses docked in a similar orientation to ATP were utilized for further analysis. Free energies (measured in kcal/mol from Schrödinger-Maestro (v. 2018–1)) of the protein, ligand, and protein-ligand complex structures were calculated using a molecular mechanics/generalized Born surface area (MM-GBSA) approach. For protein-ligand complex free energy calculations, all poses from docking were utilized. Ligand efficiencies (LEs) were calculated by averaging all docked poses binding free energies (kcal/mol) from AutoDock Vina [41] and dividing by the number of heavy atoms in the molecule. Distance measurements determined through fingerprinting were split into three different interaction types depending on the heavy atom type involved and distance: electrostatic interactions were 3.0 – 5.0 Å and involved polar atoms with partial charges, hydrophobic interactions were 3.0 – 5.0 Å and involved carbon atoms, and hydrogen bond interactions were 2.8 – 4.0 Å between hydrogen bond donor and acceptor atoms. All poses were visually assessed in PyMOL [39] and UCSF Chimera [38] to determine the binding cavity’s hit rate in position and occupancy.

Schrödinger-Maestro [39] was utilized for residue-specific interactions between the protein and docked molecules to further analyze the docking results. The protein structures were pre-processed using the Schrödinger-Maestro [39] protein preparation wizard. Using the Discovery Informatics and QSAR feature, a fingerprint of the interactions between the protein and ligand was produced, examining electrostatic, hydrophobic, hydrophilic, and backbone interactions.

Sequence alignment was performed with Schrödinger-Maestro [39] using its multiple sequence alignment feature to identify conserved residues in the three homologs essential for ligand binding in the kinase domain. The interaction fingerprints were then overlaid with the three-dimensional locations of the residues.

### Pharmaceutical ADME qualities and identification of similar FDA-approved therapeutics

FDA-approved drugs with known potential as therapeutics, that contained drug-like absorption, distribution, metabolism, and excretion (ADME) characteristics and with chemical characteristics similar (≥60% similarity) to the known ERK8 inhibitors AZ960 and Ro318220 were identified using the QikProp feature in Schrödinger-Maestro. We tested each drug by docking and evaluating it, like we did AZ960 and Ro318220, as described in the molecular docking section above.

### Purification of recombinant TbERK8 from Escherichia coli

*Escherichia coli* strain Rosetta 2 (DE3) (Cat# 71397, MilliporeSigma, Burlington, MA) was transformed with the plasmid pGEX2T expressing the full-length hERK8 coding region or pGSTAg expressing the full-length TbERK8 coding region [19,20]. One liter of Terrific Broth media containing 100 μg/ml Ampicillin and 30 μg/ml chloramphenicol in 2 L baffled flasks was inoculated with 25 mL from an overnight culture of Rosetta 2 strains harboring either the pGEX2T-hERK8 or pGSTAg-TbERK8 plasmid. Two baffled flasks were inoculated with each overnight strain and incubated and shaken at 175 RPM in a New Brunswick Innova 44R Incubator Shaker (Fischer Scientific, Pittsburgh, PA) at 37 °C until the cultures reached an O.D._600_ of 0.94. Once the cultures in the flask reached the target O.D._600_, the flasks were cooled to 4 °C in an ice water bath. The cooled cultures were induced by adding isopropyl β-D-1-thiogalactopyranoside (IPTG) to a final concentration of 0.1 mM and shaken at 16 °C for 16 hours at 175 RPM in a New Brunswick Innova 44R Incubator Shaker (Fischer Scientific, Pittsburgh, PA). On the next day, the cultures were pelleted by centrifugation at 9000 x*g* in a JLA 10.500 rotor (Beckman Coulter; Brea, CA), and the media was decanted. The pellet was flash-frozen in liquid nitrogen and then thawed on ice. The thawed pellet was resuspended in lysis buffer (25 mM Tris, 75 mM NaCl, 0.5% TritonX-100 and 0.5% Nonidet P-40, 1 mM phenylmethanesulfonyl fluoride, 1 mM benzamidine⋅HCl) and lysed using a microfluidizer M-110P from Microfluidics (Westwood, MA). The crude lysates were clarified by centrifugation at 140,000 x*g* in a 45Ti rotor (Beckman Coulter, Brea, CA) for 30 min at 4°C. The clarified lysate was loaded by gravity to a 3 mL glutathione agarose resin column (Cat# G-250–10, GOLDBIO, St. Louis, MO) formed in a 1.5 × 20 cm glass chromatography column (Cat# 7371522, Bio-Rad, Hercules, CA). The column was washed with 30 mL of PBS (pH 7.4) containing 1 mM DTT. Elution buffer (50 mM Tris buffer pH 8.0 containing 10 mM reduced glutathione and 1 mM DTT was added to the washed column), and 1 mL fractions were collected by gravity and examined by SDS-PAGE stained by Coomassie dye. Peak fractions pooled from the elution were dialyzed (4x 1:500 dilutions) in kinase buffer (30 mM Tris, pH 7.4, 10 mM MgCl_2_, 1 mM DTT, 5% glycerol).

### Kinase assays

We performed kinase assays in buffer K (30 mM Tris pH 7.4, 10 mM MgCl_2_, 1 mM DTT, 5% glycerol, 50 μM ATP, and 0.1 mg/mL BSA) at 30°C for 30 min in 30 μL reactions. GST-tagged HsERK8 and TbERK8 were tested using myelin basic protein (MBP) (Cat# 13–110, Millipore, Darmstadt, Germany) as the substrate at increasing concentrations to determine the optimal activity for the assay. ATP consumption was determined by adding equal volumes of the kinase assay reagent (Promega, Cat# V5601, Madison, WI) following the manufacturer’s protocol. Luminescence was measured using the microplate reader (SpectraMax L, Molecular Devices, Sunnyvale, CA).

### ERK8 compound screens

Idarubicin (I1656-10mg), fludrocortisone (F6127-1g), prednisolone (P6004-1g), and sildenafil (Phr1807) were purchased from MilliporeSigma (Burlington, MA). However, licensure and cost restrictions prevented famotidine and fluprednisolone from being tested experimentally in this study. These compounds were screened for kinase inhibition utilizing a luciferase-based assay. Briefly, the kinases were tested using MBP as the substrate at a 14-fold molar excess in 384-well flat-bottom white plates (BRANDTECH Scientific, Cat# 781681, Essex, CT). These compounds were tested at concentrations ranging from 10 mM to 150 nM. The kinases plus MBP substrate reactions were incubated with each inhibitor for 30 minutes at 30°C. Equal volumes of kinase assay mix Promega (Cat# V5601, Madison, WI) were added to the kinase/inhibitor reaction after 30 minutes, following the manufacturer’s protocol. ATP luminescence was measured using a microplate reader (SpectraMax L, Molecular Devices, Sunnyvale, CA). IC_50_ values were calculated from assays done in triplicate by fitting the data using Prism 8.1.1 (GraphPad).

## Results

### Sequence alignment and homology modeling of HsERK8 and TbERK8 orthologues

Each structure showed favorable psi/phi backbone angles, positive energy scores, and scored similarly to X-ray resolved structures (S1 Fig, S2 Fig and, S3 Fig). Multiple Sequence alignments performed using Schrödinger-Maestro showed that residues (Val27, Lys42, Asn55, and Asp155) in the ATP binding pocket were conserved in the ERK8 orthologs tested in this study (S4 Fig).

The kinase domains of TbERK8 and HsERK8 [19,24] were 72% similar and 51% identical in sequence. Multiple sequence alignment revealed that TbERK8 lacked residues Asp69 and Lys105. The MOE models showed that the MAPK structure from *Cryptosporidium parvum* (PDB: 3oz6), which is an ERK family member [43], shared 51% and 46% identity to TbERK8 and HsERK8 (S5 Fig and S1 Table). The key residues in the active site of Fus3, TbERK8, and HsERK8 that interact with ATP were identified and renumbered for referencing purposes in S2 Table.

The overlays of the ATP-binding site for the ERK8 ortholog showed that residue Leu19 was conserved between the ERK8 orthologs from the protists *T. brucei* and yeast. The TbERK8 ortholog substituted residues Glu95 for Met95 and Asn142 for Ser141 (S6A Fig). HsERK8 substituted the residue Gln21 for Leu19, but the residues Ser141 and Met95 were conserved in HsERK8 and Fus3 (S6B Fig). There was no conserved similarity in the other residues within the catalytic pockets of these orthologs.

**Fig 1:**
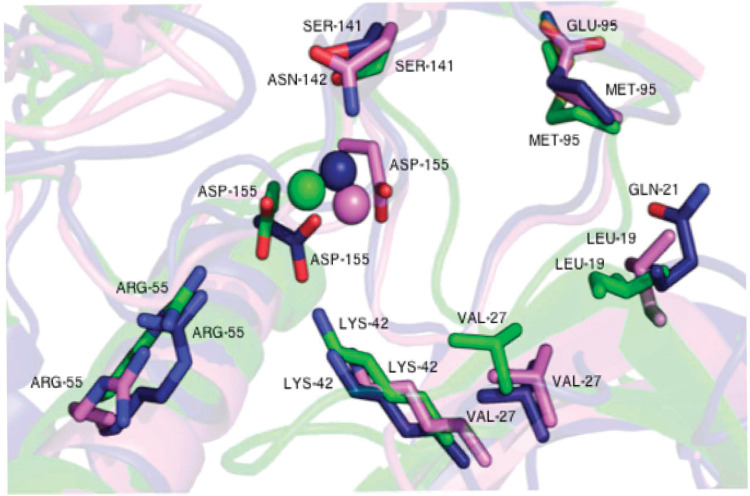
Structural features of ERK8 orthologues. Key residues of the ERK8 binding pockets are shown as stick structures, labeled and colored based on orthologue (Fus3 (green), HsERK8 (navy), and TbERK8 (violet). Spheres indicate Mg^2+^ ions.

The structural differences between TbERK8 and HsERK8 were observed for residues Leu 19, Met 95, Ser141, and Asp155 (using HsERK8 numbering). They were most notable at TbERK8 residue Asp155 positioned near the Mg^2+^ ion (Fig 1 and S6C Fig), which was shown experimentally to be essential for HsERK8 activity [44] and TbERK8 (S7 Fig). The change in the position of Asp155 in TbERK8 compared to Asp155 of HsERK8 in the superimposed models is likely due to the deletions of residues Asp69 and Lys 105. The spatial shifts did not affect ERK8 function because in enzymatic assays, both kinases bound and hydrolyzed ATP independently of the Asp69 and Lys105 residues or the Asp155 shift. This observation is consistent with the biological relevance of aspartate residues coordinating the Mg^2+^ ion in kinases and promoting catalytic events [45,46].

**Fig 2:**
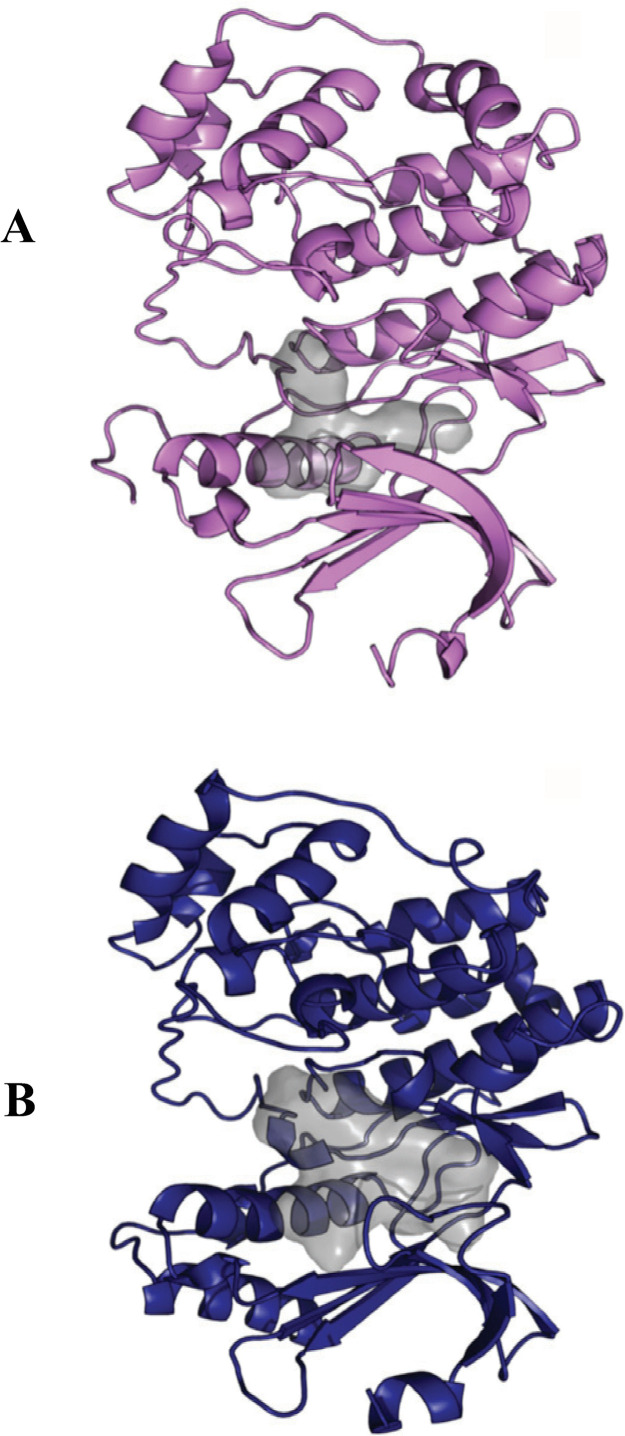
ERK8 orthologue binding cavity volume and physicochemical features. (A, B) Predicted binding pocket volume surface representation. (A) TbERK8 is shown as violet cartoon. (B) HsERK8 is shown as navy cartoon.

MetaPocket 2.0 and UCSF Chimera predicted different ATP-binding pocket shapes for each of the ERK8 orthologs. Fus3 had a large oval-shaped ATP-binding pocket with a single lobe where the ATP molecule aligned lengthwise (S8A Fig and S8B Fig). TbERK8 had an L-shaped ATP-binding pocket with a volume of approximately 520 Å^3^ in which the ATP molecule fit neatly into the long side of the pocket (Fig 2A, S8C Fig, and S8D Fig).

The HsERK8 ATP-binding pocket was more extensive, with two asymmetric shallow lobes and an estimated volume of 1186 Å^3^. The ATP molecule fits the length of the HsERK8 pocket, and the γ-phosphate bends toward the shorter lobe (Fig 2B, S8E Fig, and S8F Fig).

**Fig 3:**
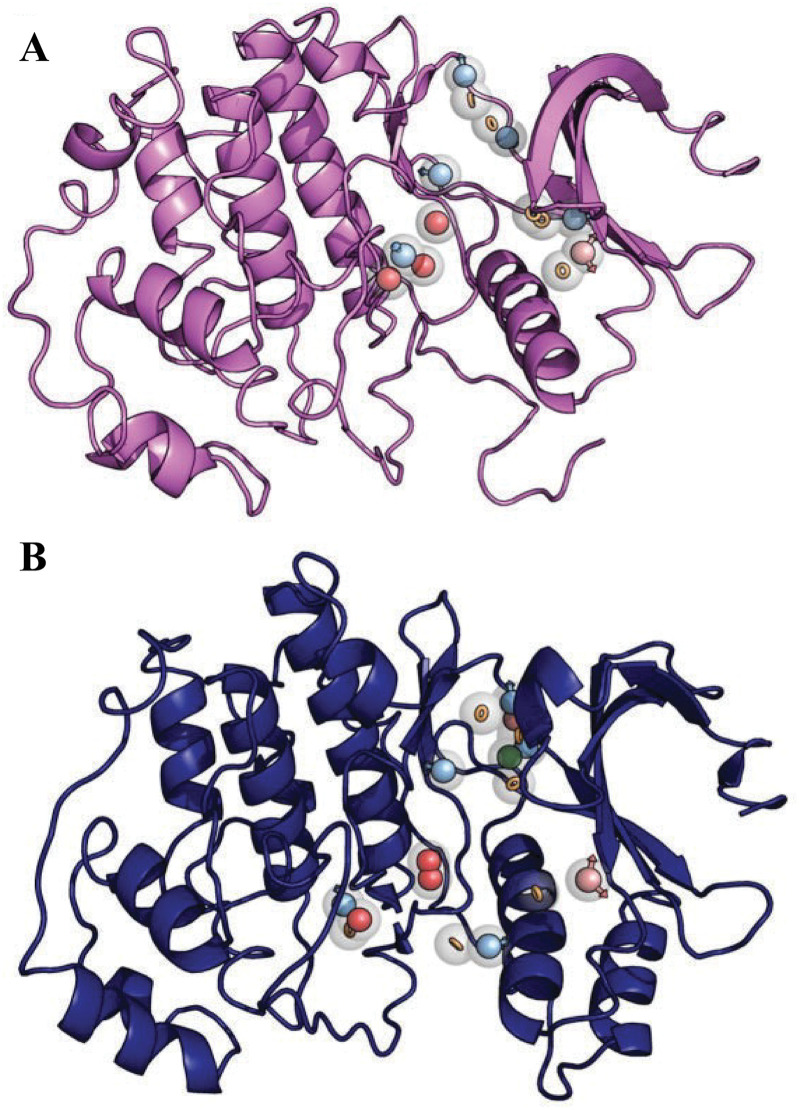
Pharmacophore model of TbERK8 and HsERK8. (A) TbERK8 is shown as violet cartoon, and (B) HsERK8 as navy cartoon. Spheres represent the pharmacophore model features: red circles - negative ionic, blue sphere with arrows - hydrogen bonding donor, pink sphere with arrows - hydrogen bonding acceptor, green sphere - hydrophobic, and orange rings - aromatic features.

Pharmacophore models of TbERK8 (Fig 3A) and HsERK8 (Fig 3B) had charged features that matched their respective surface maps. Moreover, these features were organized in spatially different positions within the binding pocket, potentially influenced by amino acid insertions and deletions. However, both orthologues accommodate the negatively charged triphosphate binding region, allowing kinase activity.

### Molecular docking and interaction analysis

**Fig 4:**
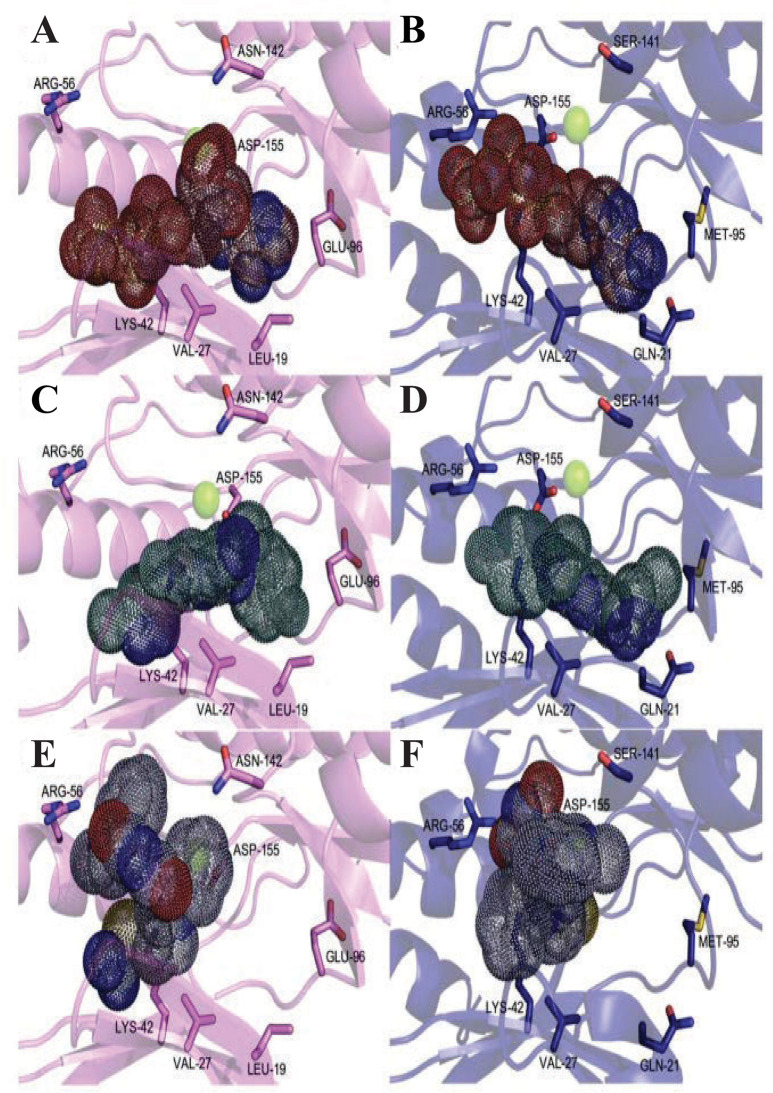
Volume representation of known orthologue-specific best inhibitor poses for TbERK8 and HsERK8. (A) TbERK8 and ATP, (B) HsERK8 and ATP, (C) TbERK8 and AZ960, (D) HsERK8 and AZ960, (E) TbERK8 and Ro318220, and (F) HsERK8 and Ro318220. TbERK8 is shown as violet cartoon, and HsERK8 as navy cartoon with key residues as stick figures colored by atom type—the Mg^2+^ ion is shown as a green sphere. ATP is represented in volumetric dots colored by atom type with maroon carbon atoms. AZ960 is shown as volumetric dots colored by atom type with teal carbon atoms. Ro318220 is shown as volumetric dots colored by atom type with light blue carbon atoms.

The Fus3 co-crystal with ADP (PDB ID: 2b9f) [27], previously used as the starting model for an *in silico* HsERK8 study [24], also served as the template for the TbERK8 docking studies. Re-docking of ADP into Fus3 (S9 Fig and S3 Table) resulted in a root mean square deviation (RMSD) of 0.710 Å, less than the 2.0 Å benchmark for successful re-docking studies [47]. These RMSD values validated the box size and position for all docking experiments. ATP interactions with Val27, Lys42, and Asp155 were conserved for all three orthologs. The top nine ATP poses for Fus3 (S10A Fig), TbERK8 (S10B Fig), and HsERK8 (S10C Fig) had the lowest energy scores and domains positioned appropriately for autophosphorylation, with the phosphate groups coordinated near the Mg^2+^ ion. Molecular docking predicted that ATP, having a volume of 348.3 Å^3^, could fit in the binding pockets of TbERK8 (Fig 4A) and HsERK8 (Fig 4B). Removing the γ phosphate from ATP resulted in RMSD values for ATP, relative to the ADP position in the crystal structure of Fus3, of 2.028 Å, 1.811 Å, and 1.920 Å for Fus3 (S11A Fig and S4 Table), TbERK8 (S11B Fig and S4 Table), HsERK8 (S11C Fig and S4 Table), respectively. The ERK8 orthologs had conserved structures that, when overlaid, with the ATP molecules assuming different positions in each pocket (S11D Fig and S4 Table). The RMSD values of ATP, relative to the ADP position in the crystal structure of Fus3, indicate that the protocol can account for larger ligands and provide useful information for interactions between proteins and small molecules.

A previous study showed that TbERK8 and HsERK8 had different inhibitor profiles [20], best explained by fundamental differences between their ATP-binding pockets. The molecular docking analyses in this study identified structural determinants responsible for ortholog selectivity between Ro318220 and AZ960. Both Ro318220 and AZ960 contained pyrrole-dione and methyl-sulfenyl moieties. They also contained electropositive and electronegative regions (S5 Table). Ro318220 displayed hydrophobic features, hydrogen bond donors, and acceptors, whereas AZ960 exhibited hydrogen bond donors, aromaticity, and hydrophobicity features. S5 Table lists the volumes of selected compounds, showing that idarubicin (402.8 Å^3^) and sildenafil (423.1 Å^3^) were similar to that of Ro312880 (404.0 Å^3^). They displayed regions of concentrated positive charge. Famotidine was relatively small, with a volume of 251.0 Å^3^, and displays a patch of surface area with a net negative charge. Prednisolone, fluprednisolone, and fludrocortisone had analogous structures and were similar in size to AZ960, although fludrocortisone lacked a potential hydrogen-bonding carbonyl oxygen that was present in both prednisolone and fluprednisolone. They mainly had neutrally charged surfaces and volumes ranging from 325 to 350 Å³.

The ligand pharmacophore model generated for Ro318220 (S12A Fig) and AZ960 (S12B Fig) identified hydrogen bond acceptor features. The molecular docking analyses predicted that the TbERK8 ATP-binding pocket can readily accommodate AZ960 or Ro318220 with volumes of 292.5 Å^3^ and 404.0 Å^3^, respectively (Fig 4C and 4E and S5 Table). The larger ATP-binding pocket for HsERK8 also easily accommodated AZ960 and Ro318220 (Fig 4D and 4F and S5 Table).

Seven out of nine Ro318220 poses docked into TbERK8 (S13A Fig) resulted in partial solvent exposure of the electronegative pyrrole-dione moiety region, including the best overall pose (S13B Fig). Docking of Ro318220 with TbERK8 showed fewer common binding positions (S6 Table). Ro318220 showed more common positions in HsERK8 (S6 Table), with none of its best poses exposed in the solvent (S13C Fig and S13D Fig). Docking analysis for AZ960 showed common binding positions throughout all nine poses with TbERK8 (S14A and S14B Fig and S7 Table), while the nine docking positions for HsERK8 were less consistent (S14C and S14D Fig and S7 Table).

**Fig 5:**
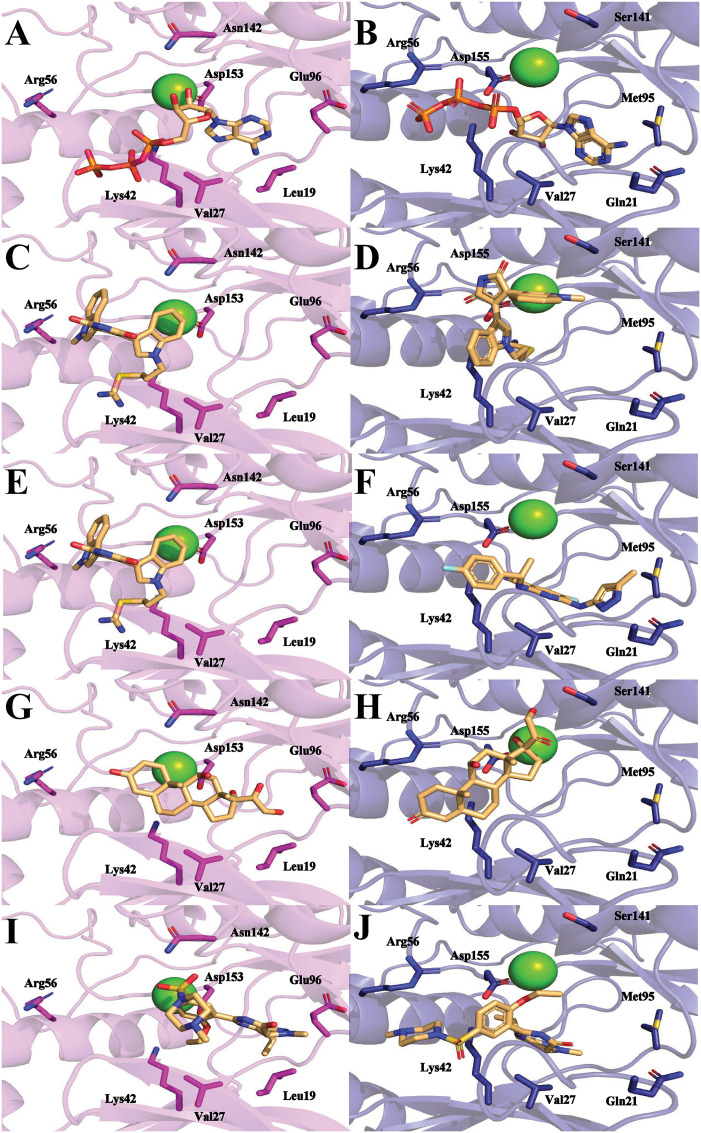
Known orthologue-specific inhibitors docked into TbERK8 and HsERK8. (A) TbERK8 and ATP, (B) HsERK8 and ATP, (C) TbERK8 and Ro318220, and (D) HsERK8 and Ro318220, (E) TbERK8 and AZ960, (F) HsERK8 and AZ960, (G) TbERK8 and prednisolone, (H) HsERK8 and prednisolone, (I) TbERK8 and sildenafil, (J) HsERK8 and sildenafil. In all panels, TbERK8 is shown in violet cartoon, and HsERK8 is shown in navy cartoon, with key residues in sticks colored by atom type. Mg^2+^ is shown as a green sphere. In every panel the docked small molecules are as sticks colored by atom type with carbons in beige.

Docking studies in S8 Table, which use single-letter codes, indicate that ATP interacts with TbERK8 at key residues, including Leu19, Val27, Lys42, Asn142, and Asp155, with interatomic distances predicted to be less than 5.0 Å. Fig 5A shows the model of such interactions. ATP interactions with HsERK8 occur at key residues (Val27, Lys42, Arg56, Gln21, and Asp155) with distances less than 5.0 Å; however, for Met 95, the distance is greater than 5.0 Å (S8 Table and Fig 5B).

The docking studies with Ro318220 showed stronger electrostatic and hydrophilic interactions for HsERK8 than for TbERK8 (S8 Table). For TbERK8, S8 Table and the docking model in Fig 5C predicted that Arg56 and Val27 were the only residues having interatomic distances less than 5.0 Å to Ro318220. The residue Lys42 had a large interatomic distance of 5.0 Å.

For HsERK8, the predicted key interactions with Ro318220 occurred at electrostatic residues Lys42, Arg56, Glu60, and Asp155, as well as the hydrophobic residue Val27. In the HsERK8 docking model, all these key residues had interatomic distances less than 5.0 Å (S8 Table and Fig 5D). The interactions of Ro318220 with key residues Val27, Lys42, Arg56, Glu60, and Asp155, plus additional interactions with the charged residue Lys139 and hydrophilic residue Ser141 in Tabe S8 show that it has the chemical features and size required to bind competitively against ATP in HsERK8. Ro318220 does not compete well with ATP in the TbERK8 binding pocket, but it provides a plausible model to explain the selectivity of Ro318220 for HsERK8. These *in silico* observations suggest that the ability of AZ960 or Ro318220 to form close interactions with Lys42, with bond distances less than 5.0 Å, is critical for them to inhibit TbERK8 or HsERK8 activity.

The key interactions between AZ960 and TbERK8 occurred at residues Lys42, Asp153, and Arg56 (S8 Table). The docking models we generated in Fig 5E predicted that only Val27 and Lys42 had interatomic distances less than 5.0 Å to the ligand. The HsERK8 and AZ960 docking studies suggested that the key interactions occurred between residues Lys42, Glu60, Asp155, Gln21, and Val27 (S8 Table). However, the model we generated in Fig 5F for HsERK8 and AZ960 did not predict a role for Glu60 interacting with AZ960. This HsERK8 model indicated that residues Asp155 and Gln21 were the only ones with interatomic distances less than 5.0 Å. The S8 Table results also suggested that the distance between Lys42 and the ligand would be greater than 5.0 Å.

The overlayed homology models indicated that AZ960 aromatic interactions occurred differently in the TbERK8 and HsERK8 orthologues (S15A Fig). AZ960 had a π-stacking interaction with Phe156 at 3.6 Å with TbERK8 (S15B Fig) but did not interact with the corresponding residue in HsERK8. This π-stacking interaction may reflect the shift in residues caused by the two residue deletions (Asp69 and Lys105) in TbERK8. Although residue Phe156 in HsERK8 did not interact with AZ960, a potential π-stacking interaction was observed with Phe97 in the upper and back-end of the adenine binding region, away from the key residues (S15C Fig). It may have influenced a feature reorganization, making these aromatic components more accessible for small-molecule binding in TbERK8 (S15D Fig). The homology models suggest that the spatial orientation of residue Phe156 could drive ATP competition with AZ960 in TbERK8.

### Probing ERK8 binding sites for inhibitor specificity by molecular docking

The Schrödinger-Maestro QikProp tool selected famotidine (S16 Fig and S9 Table), fludrocortisone (S17 Fig and S10 Table), fluprednisolone (S18 Fig and S11 Table), idarubicin (S19 Fig and S12 Table), prednisolone (Figs 5G, 5H, and S20 and S13 Table), and sildenafil (Figs 5I, 5J, and S21 and S14 Table) as the top six small-molecule pharmaceutical compounds with physicochemical features most similar to Ro318220 and AZ960. Ro318220 was similar to idarubicin (63.77%), famotidine (62.99%), and sildenafil (60.08%). AZ960 was similar to prednisolone (64.68%), fludrocortisone (63.59%), and fluprednisolone (63.44%). We used these six molecules for docking studies against HsERK8 and TbERK8.

S15 Table lists the interactions between the compounds when docked into HsERK8 and TbERK8. Idarubicin adopted a pose similar to Ro318220, notably having electrostatic interactions with the key residues Lys42 and Asp155 in HsERK8 while also displaying hydrophobic interactions. Idarubicin interacted similarly to TbERK8 with key residues Lys42 and Asp155 and encompassed molecular interactions deeper in the binding cavity of TbERK8, which was absent in Ro318220. Sildenafil had electrostatic interactions with the residues Lys42, Glu60, and Asp155 in the docking studies with HsERK8. The TbERK8 docking study predicted that residues Lys75, Glu93, Glu96, Asp97, and Asp155 within its ATP-binding pocket interacted with sildenafil, presumably electrostatically, despite its larger molecular volume. Here, we observed that TbERK8 residues Lys75, Glu93, Glu96, and Asp97 made up unique, apparently electrostatic interactions with sildenafil that resulted in a + 2 difference in charge compared to those between HsERK8 and sildenafil.

These docking results suggest that this larger flexible molecule with negative electrostatic features that drive interactions deep within the binding cavity would be optimal for both ERK8 orthologs.

Famotidine docking results did not show similar binding interactions to HsERK8 as Ro318220, with only one electrostatic interaction and most of the interactions being hydrophobic.

When docked into both ERK8 orthologues, prednisolone, fluprednisolone, and fludrocortisone displayed similar interactions within each orthologue, mostly backbone hydrogen bonding and hydrophobic interactions driving the binding. Prednisolone had apparent electrostatic interactions with residues Lys42, Lys139, and Asp155 in HsERK8 and apparent electrostatic interactions with residues Lys75, Glu96, and Asp155 when docked into TbERK8. There were no differences between net charges of the HsERK8 or TbERK8 residues that made apparent electrostatic interactions with prednisolone. However, the interaction between HsERK8 and prednisolone involved the critical residue Lys42.

Docking showed the apparent electrostatic interactions between the protein and fludrocortisone occurred with residues Lys42, Lys139, and Asp155 in HsERK8. The apparent electrostatic interactions between TbERK8 and fludrocortisone occurred at residues Lys75, Glu96, and Asp155. There were no differences between the net charges of the HsERK8 or TbERK8 residues that made apparent electrostatic interactions with fludrocortisone. However, the interaction between HsERK8 and fludrocortisone involved the critical residue K42. Fluprednisolone interacted with the key residues Lys42 and Asp155 in HsERK8 but not with any of the key residues in TbERK8.

### Screening ERK8 orthologs with top compounds from docking studies

Molecular docking analyses indicated that sildenafil, prednisolone, idarubicin, and fludrocortisone were the top candidates that would act as ERK8 ortholog-specific inhibitors. Table 1 compares the potency of each FDA-approved agent for inhibiting purified GST-tagged HsERK8 (S22A Fig) and TbERK8 (S22B Fig) overexpressed in *E. coli*. It shows that prednisolone was an ortholog-specific inhibitor of HsERK8 (S22C Fig). The very high IC_50_ of prednisolone for TbERK8 compared to HsERK8 may be due to an overestimation of molecular flexibility by the docking model, as its volume is comparable to AZ960.

**Table 1:** FDA-approved drugs tested in kinase inhibition assays.

Compound	Structure	TbERK8			HsERK8		
		IC_50_ [mM]	95%CI [mM]	Goodness of fit [R^2^]	IC_50_ [mM]	95%CI [mM]	Goodness of fit [R^2^]
Prednisolone	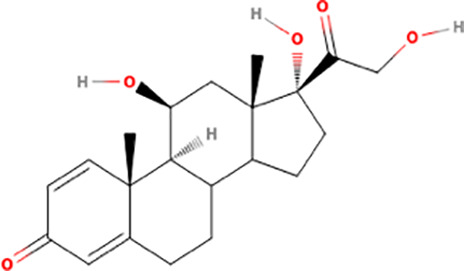	>10	Too wide	0.9677	0.3	0.15 - 0.5	0.9431
Sildenafil	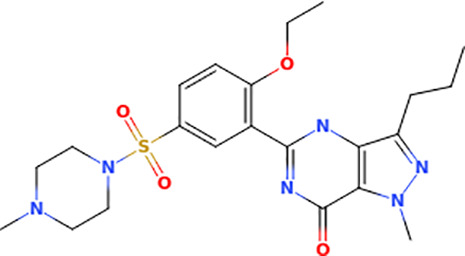	9	5 - 18	0.9743	8	5 - 12	0.967
Idarubicin	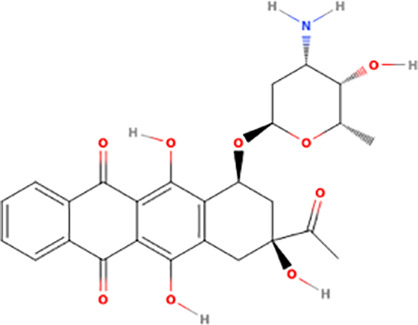	n/d	-	-	n/d	-	-
Fludrocortisone	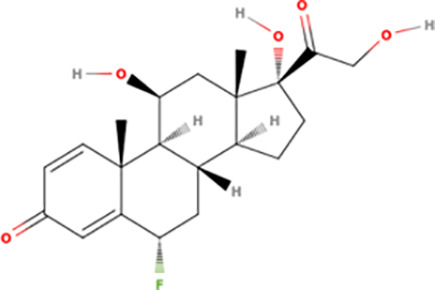	n/d	-	-	n/d	-	-

However, sildenafil is a weak inhibitor, so clear conclusions about which factors drive binding cannot be drawn.

Sildenafil, shown in Table 1, had a low potency for inhibiting TbERK8 and HsERK8 IC_50_ with essentially identical values (S22D Fig). An electrostatic interaction with the key residue Asp155 was the only one conserved between TbERK8 and HsERK8 in docking studies with sildenafil (S15 Table). These docking studies indicate that the Asp155 electrostatic interaction was critical for sildenafil inhibitor potency, supported by essentially identical IC_50_ values of sildenafil for HsERK8 and TbERK8.

Idarubicin was a deep red compound at concentrations above 4.0 x10^-5^ M, which blocked the luminescence signal of the assay. At concentrations below 4.0 x10^-5^ M, idarubicin had no detectible (n/d) inhibitory effect on the kinase activity of TbERK8 or HsERK8 (Table 1). The docking model did not predict the inert nature of idarubicin for TbERK8 or HsERK8, making it a good experimentally tested decoy molecule for future molecular docking studies [48].

The compound fludrocortisone did not inhibit the kinase activity of TbERK8 or HsERK8 at any tested concentration. The docking model did not predict the inability of fludrocortisone to inhibit either ERK8 homolog, suggesting that its small volume was insufficient for it to interact simultaneously with all parts of the binding pockets for either ortholog.

### *Docking with non-specific* inhibitor of *ERK8 orthologs*

AZD5438 is a potent inhibitor of TbERK8 and HsERK8 orthologues [19], even though its volume (357.8 Å³) is intermediate between AZ960 and Ro318220. The docking of AZD5438 (S15 Table) showed that it interacted with two key residues in HsERK8: Val27 and Lys42 (S15 Table). AZD5438 interacted with four of TbERK8’s key residues: Leu19, Val27, Lys42, and Asp155 (S15 Table). Additionally, it interacted with Phe156, which influenced the docked spatial placement of AZ960. According to the docking model, AZD5438 interacts with key residues in the triphosphate binding regions for both ERK8 orthologs and the adenine binding region of TbERK8, which is otherwise less accessible. The intermediate volume of AZD5438 is consistent with its potential for potent inhibition of both TbERK8 and HsERK8 [19].

The TbERK8 residue Lys75 does not interact with AZ960 or Ro318220 in the docking models. However, Lys75 appeared to contribute to the binding of AZD5438 in TbERK8 through electrostatic interactions (S15 Table). The smaller ATP binding pocket of TbERK8 did not accommodate the larger molecule Ro318220 or the smaller molecule prednisolone. However, the negatively charged moieties in the flexible domain of sildenafil likely enable it to inhibit TbERK8 despite its volume being larger than that of Ro318220. The docking results support the idea that the negatively charged moieties of sildenafil help it access the TbERK8 small ATP-binding pocket. Thus, residue Lys75 plays a role in sildenafil inhibiting TbERK8 and represents an exploitable residue for attracting ortholog-specific compounds.

In HsERK8, the larger adenine binding pocket can easily accommodate sildenafil, which has a volume greater than Ro318220. Ile74 leads to a more hydrophobic region within the larger adenine binding pocket of HsERK8 in the three-dimensional model. Of the TbERK8 putative ligands, AZ960 had the best ligand efficiencies (LE = 0.264 ± 0.005 kcal/mol), while the worst was Ro318220 (LE = 0.161 ± 0.018 kcal/mol) (S23A Fig) [20]. AZ960 had the best LE (LE = 0.290 ± 0.010 kcal/mol) for HsERK8, while Ro318220 scored second to last (LE = 0.194 ± 0.012 kcal/mol) (S23B Fig). When these data were organized by molecular volume, a trend was apparent for TbERK8 ligands (S23C Fig); the smaller TbERK8 cavity volume limited the size of ligand. The significant difference in binding pocket size between TbERK8 and HsERK8 made LE a valuable metric for ranking compounds with known experimental data. However, docking compounds of similar size in the binding pocket of a single ERK8 ortholog gave the best method for ranking compounds for their selectivity with a ligand pharmacophore model.

Based on docking studies, a dihydroxycyclopentyl-ethanone moiety in prednisolone interacts with residue Lys75, a unique structural feature in TbERK8. A model incorporating this feature into the scaffold of AZ960 is suggested to improve its ligand selectivity for TbERK8 over HsERK8. This model led to compound **1** being the top potential TbERK8 selective inhibitor based on its low volume and high number of rotatable bonds (S16 Table).

*In silico* alterations of the compound **1** scaffold led to six additional analogs containing hydrogen bond-accepting features that could interact with TbERK8 residue Lys75 (S16 Table). Compounds **5** and **6** had the smallest differences in calculated binding energies between TbERK8 and HsERK8 (S17 and S18 Tables) and followed similar binding patterns to those of the known ERK8 inhibitors AZ960 and AZD5438.

When evaluating protein-small molecule interactions, two compounds were predicted to bind more effectively to TbERK8 than to HsERK8: compounds **5** and **6,** most likely because of backbone interactions (S19 Table). These compounds’ interactions with residue Asp98 of HsERK8 were like those of AZD5438, and they had hydrophobic and backbone residues comparable to those of AZ960. The interactions of compounds **5** and **6** with TbERK8 resembled those of AZD5438 rather than AZ960, possibly due to interactions between the compounds and residue Thr57 (S19 Table). The LE calculations predicted that compound **6** (S24A Fig) could be an interesting candidate with properties that allow it to bind broadly to HsERK8 and TbERK8.

The MM-GBSA free energy calculations using Schrödinger-Maestro showed that AZ960 and AZD5438 had measurably better, more negative binding energies for TbERK8 (-107.97 ± 11.24 kcal/mol and -109.44 ± 11.76 kcal/mol, respectively) compared to HsERK8 (-98.60 ± 10.83 kcal/mol and -96.00 ± 14.95 kcal/mol, respectively) (S24B Fig). These results were consistent with previous experimental data [19]. Ro318220, however, did not yield the expected free energy values, ultimately having less favorable predicted binding energies in both TbERK8 and HsERK8, which may be influenced by protein dynamics not accounted for in this work, as well as the large molecular size and pocket volume of HsERK8.

**Fig 6:**
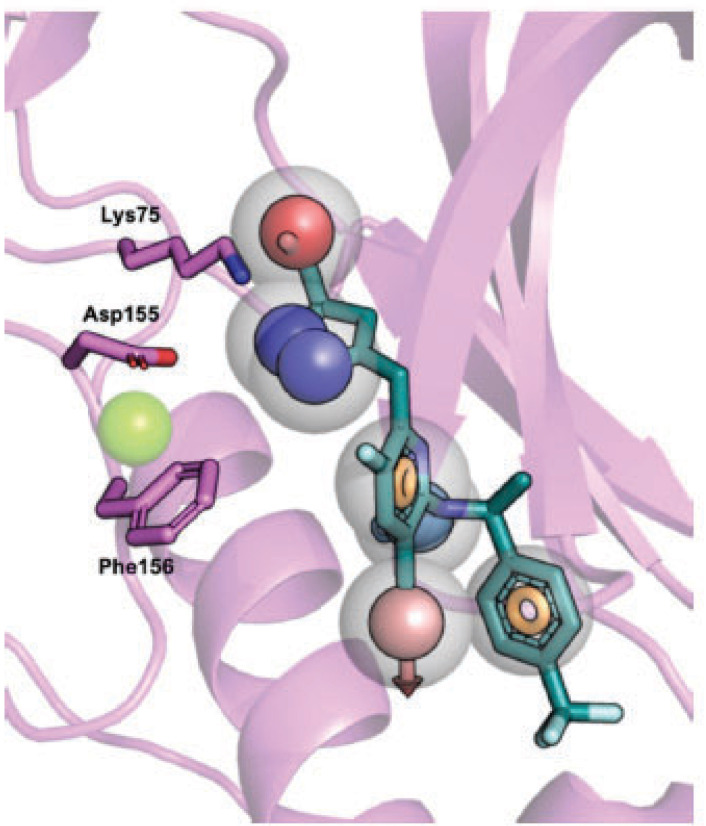
Proposed characteristics for TbERK8-specific inhibitors. These include having a small and flexible molecular scaffold, an aromatic core for interactions with Phe156, and polar flanking tails to interact with Lys75 and the triphosphate binding region.

The pharmacophore model derived from the docking results (Fig 6) indicates that the TbERK8 ATP-binding pocket featured more charged and aromatic moieties. Residue Lys75 in TbERK8 occupied a larger proportion of the binding pocket than Ile74, which is in an equivalent spatial position in the HsERK8 model, reducing the overall pocket volume of TbERK8 and increasing the net positive charge in the adenine binding region of the TbERK8 active site. Therefore, we hypothesize that compounds **5** and **6** would be good leads for discovering molecules that are selective inhibitors for TbERK8 but not HsERK8, providing a buildable scaffold for next-generation compound design and rationale.

## Discussion

All *T. brucei* therapeutics were discovered using phenotypic screening approaches except for eflornithine, which targets ornithine decarboxylase and polyamine synthesis [49]. The phenotypic screening approach for drug discovery has been a more productive and successful strategy [50,51]. However, antitrypanosomal drugs developed by this strategy have suffered from poor efficacy and toxic side effects.

The major weakness of using a target-based approach for discovering antitrypanosomal agents is the overall inefficiency of this process. Less than ten percent of FDA drugs were discovered by using a target-based strategy [51]. However, we previously combined a target-based screen with a phenotypic screen that discovered AZ960 bioactivity against *T. brucei* and its potency and selectivity for inhibiting TbERK8 [20]. While there is no experimental structure of this protein, we do have a human-curated homology model [24], which, while not built by modern large language model computational methods, has been rigorously vetted by expert analysis. The fact that some of the model statistics for this specific TbERK8 model were suboptimal may have contributed to the inability to find a TbERK8-specific ligand. This study increased the likelihood of success for discovering TbERK8 ortholog-specific inhibitors using an *in silico* approach. Identifying prednisolone, the FDA-approved corticosteroid used to reduce inflammation, as an ortholog-specific inhibitor for HsERK8 validated our *in silico* approach. The IC_50_ of prednisolone for HsERK8 is insignificant compared to 5–10 nM reported for the HsERK8-specific inhibitor Ro318220 [44]. Likewise, the IC_50_ of sildenafil for TbERK8 is insignificant compared to the published 120 nM ± 5 for the TbERK8-specific inhibitor AZ960 [20]. Therefore, a limitation of this *in silico* approach was its apparent inability to discover ERK8 inhibitors with IC_50_ values in the nanomolar range. Such limitations are not uncommon outcomes in virtual screening nor in high-throughput screening or other kinds of drug discovery campaigns [52]. However, this *in silico* approach predicted prednisolone and sildenafil as two compounds from an FDA library that can use ortholog-specific mechanisms for inhibiting HsERK8 and TbERK8. This approach gave a successful result and serves as a good proof-of-principle for this methodology. It also provides a starting point for more detailed discovery and improvement efforts, as the scaffolds of these two compounds can be modified in future medicinal chemistry campaigns to improve their IC_50_ values.

Furthermore, our prediction that sildenafil would inhibit TbERK8 through interactions with residue Lys75, through its apparent electrostatic role in ligand binding, was also consistent with our experimental results. We have additionally suggested that compounds **5** and **6** will be novel leads for optimization, thereby enhancing their specificity and potency against TbERK8. These compounds are analogs of AZ960, a compound in preclinical development [53], and represent buildable scaffolds for expanding the repertoire of antitrypanosomal agents. Their optimized scaffolds may be less likely to have adverse side effects caused by off-target events. Future studies with compounds **5** and **6** will combine biochemical and phenotypic assays to mitigate weaknesses associated with using a target-based approach in this study with a likely focus upon the addition of electron-donating substituents to the fluorobenzene ring (compound **5**) to enhance the pi-cation interaction between the ligand and Lys75 in TbERK8 as suggested by molecular docking.

## Conclusion

Here, we demonstrate that small-molecule inhibitors that can specifically inhibit either human or parasite ERK8 can be identified using a virtual screening protocol and model binding sites. After experimental testing, we screened four FDA-approved drugs and identified two as weak to moderate ERK8 inhibitors. Sildenafil inhibited both orthologs equally, whereas prednisolone had notably only inhibited HsERK8. The behaviors of known ERK8 inhibitors AZ960 and Ro318220 were consistent with this binding model. AZ960 specifically inhibited TbERK8 because its small volume and flexibility allowed it to fit the smaller ATP-binding pocket, including contacts with residues Lys42, Val27, and Phe156. Ro318220 preferentially inhibited HsERK8 because its large volume fits better into the large ATP-binding pocket of this kinase, allowing it to make electrostatic interactions with the key residues. Combined *in silico* and biochemical techniques suggested that residue Lys75 in the TbERK8 ATP-binding pocket alters the shape and charge of the binding surface compared to Ile74 in HsERK8. Additionally, we proposed two potential, buildable TbERK8-specific compounds, **5** and **6,** based on the AZ960 scaffold. Compounds **5** and **6** have small volumes, electronegative regions, and seven rotatable bonds, which are expected to give them significant flexibility to allow apparent electrostatic interactions with the key residue Lys75 in TbERK8.

## Supporting information

S1 Fig Energy minimized structure validation of the Fus3 crystal structure (PDB ID: 2b9f). (A) Ramachandran plot showed 95.5% of residues within the favored region, 4.2% within the allowed region, and 0.3% in the outlier region. (B) ANOLEA indicates favorable free energy scores (green). No major unfavorable residue regions were observed. (C) ProSA indicates a Z-score (-9.11, black dot) in the acceptable spectrum compared to resolved protein structures. (D) QMEAN analysis indicates mostly favorable Z-scores in solvation, SSE agreement, and all-atom interactions. Negative Z-scores were observed for torsion, ACC agreement, and CB interactions. (E) Verify3D results showed most residues above the threshold for favorable side chain placement (dotted yellow line). Residue ranges below the threshold are not within the active site.
(TIFF)

S2 Fig Energy minimized structure validation of the TbERK8 homology model (sequence accession: Tb927.10.5140). (A) Ramachandran plot showed 88% of residues within the favored region, 8.1% within the allowed region, and 3.9% in the outlier region. (B) ANOLEA indicates favorable free energy scores (green). No major unfavorable residue regions were observed. (C) ProSA indicates a Z-score (-2.7, black dot) in the acceptable spectrum compared to resolved protein structures. (D) QMEAN analysis indicated negative Z-scores in all analysis areas. (E) Verify3D results showed most residues above the threshold for favorable side chain placement (dotted yellow line). Residue ranges below the threshold are not within the active site.
(TIFF)

S3 Fig Energy minimized structure validation for HsERK8 homology model (sequence accession: Q8TD08). (A) Ramachandran plot showed 89.3% of residues within the favored region, 9.5% within the allowed region, and 1.2% in the outlier region. (B) ANOLEA indicates favorable free energy scores (green). No major unfavorable residue regions were observed. (C) ProSA indicates a Z-score (-7.87, black dot) in the acceptable spectrum compared to resolved protein structures. (D) QMEAN analysis indicated negative Z-scores in all analysis areas. (E) Verify3D results showed most residues above the threshold for favorable side chain placement (dotted yellow line). Residue ranges below the threshold are not within the active site.
(TIFF)

S4 Fig Multiple sequence alignment (MSA) of the kinase domain of ERK8 in Fus3 (PDB: 2b9f), TbERK8, HsERK8, and MAPK template (PDB: 3oz6). Alignment performed using Schrödinger-Maestro’s multiple sequence viewer. Sequences are displayed as single-letter amino acids, with conserved residues colored based on side-chain property and alignment quality. The Fus3 sequence was used as the reference, with percent similarity (S) and identity (I) indicated in the right column.
(TIFF)

S5 Fig Pairwise sequence alignment of the kinase domain of ERK8 in TbERK8 and HsERK8. Alignment performed using Schrödinger-Maestro’s multiple sequence viewer. Sequences are displayed as single-letter amino acids, with conserved residues colored based on side-chain property and alignment quality. The right column indicates percentage similarity (S) and identity (I).
(TIFF)

S6 Fig ATP binding site overlays. (A) Fus3 and TbERK8, (B) Fus3 and HsERK8, (C) HsERK8 and TbERK8, and (D) Fus3, TbERK8, and HsERK8. All structures are shown as cartoons and colored as Fus3 (green), TbERK8 (violet), and HsERK8 (navy). Residues in the ATP binding cavity are labeled and shown as stick structures and colored based on the kinase orthologue. Mg^2+^ is shown as spheres and colored based on the kinase orthologue. Sphere scale set to 0.6 Å.
(TIFF)

S7 Fig TbERK8 residue Asp155, like Lys42 is essential for normal kinase activity. Expression of TbERK8_HA_ constructs in the bloodstream form of *T. brucei.* Extracts isolated from control or tetracycline-induced *T. brucei* expressing wild-type TbERK8_HA_ were grown in a culture medium for 24 h and then prepared for examination. (A) Immunoblot assay against lysates from control (Tet-) or induced (Tet+) *T. brucei* strains that overexpress TbERK8_HA_ (Top Panel. Arrow points to 52kDa TbERK8_HA_ band recognized by α-HA). Each lane represents lysates from about 2.5 x10^6^ parasites for immunoblot loading control (Bottom Panel. Arrow points to ~55kDa VSG_221_ band in corresponding Coomassie-stained gel in loading control). (B) Immuno-precipitation (IP) and ^32^P-autoradiography of TbERK8 variants. Lysates from 10^8^ tetracycline-induced *T. brucei* strains, each overproducing TbERK8-WT_HA_, TbERK8-K42A_HA,_ or TbERK8-D155A_HA_ variants, were incubated with anti-HA antibodies for 24 h. Each lysate was incubated with Protein A agarose beads overnight and washed. The beads were incubated with kinase buffer containing 10μCi ^32^P-γ- ATP, washed, resolved by SDS-PAGE, and examined by autoradiography (Top Panel. Arrow points to the 52kDa auto-phosphorylated TbERK8_HA_ band. Variability of band size in the D155A lane may result from the reduced intensity of labeling by ^32^P-γ-ATP, indicative of its decrease in auto-phosphorylation. The variability of band size in the K42A lane likely results from kinase activity unrelated to TbERK8 captured by the IP beads, as previously observed [19]). (Bottom panel. Silver-stained SDSPAGE loading control from ^32^P-IP assay. Arrow points to ~55kDa immunoglobulin G heavy chain (IgG_HC_)) Ladder shown is GoldBio Bluestain.
(TIFF)

S8 Fig ERK8 orthologues and ATP binding site volume representation overlay with ATP. The volume pocket of the ATP binding cavity was created using MetaPocket 2.0 and calculated with UCSF Chimera. (A, C, E) The binding pocket is shown as spheres to indicate the volume and shape of the ATP binding cavity for each structure. (B, D, F) Overlays of the volume representation with ATP were also performed to highlight the orientation of ATP in the predicted pocket. All structures are shown as cartoons and colored by Fus3 (green), TbERK8 (violet), and HsERK8 (navy). ATP is shown by its stick structure and colored by element.
(TIFF)

S9 Fig ADP redocking results compared to the Fus3 X-ray crystal structure. The Fus3 structure is shown in a cartoon and colored as an X-ray crystal structure (green), and the energy-minimized structure is used for redocking (purple). The lowest energy docked pose of ADP docking results (pose 1) is overlaid with the crystal structure position of ADP. ADP is shown by its stick structure and colored by structure (X-ray crystal structure in green, re-docked pose in purple). The docked pose had an RMSD of 0.710 Å compared to the X-ray crystal structure pose.
(TIFF)

S10 Fig Results of all poses of ATP docking in ERK8 orthologues. (A) Fus3 docking results with all poses displayed as stick structures and colored by energy scores in red, orange, yellow, green, blue, and violet (ROYGBV). Fus3 is shown as a green cartoon with Mg^2+^ as a green sphere. (B) TbERK8 docking results with all poses displayed as stick structures and colored by energy scores in ROYGBV. TbERK8 is shown in violet cartoon with Mg^2+^ in green sphere. (C) HsERK8 docking results with all poses generated are displayed as stick structures and colored by energy scores in ROYGBV. HsERK8 is shown in navy cartoon with Mg^2+^ in green sphere.
(TIFF)

S11 Fig Lowest energy docked poses of ATP in ERK8 orthologues. (A) Fus3 with best pose (#1) of ATP docking results, (B) TbERK8 with best pose (#1) of ATP, (C) HsERK8 with best pose (#3) of ATP, and (D) all three structures overlaid. Fus3, TbERK8, and HsERK8 are shown in cartoon colored green, violet, and navy, respectively, with Mg^2+^ in green sphere. ATP structures are represented by their stick structure and colored by associated protein and atom type. RMSD of ATP sans γ phosphate pose compared to ADP in the Fus3 crystal structure is 2.028 Å, 1.811 Å, and 1.920 Å, respectively.
(TIFF)

S12 Fig Ligand pharmacophore model of ortholog-specific inhibitors. (A) Ro318220 (teal) and (B) AZ960 (light blue) are displayed as stick structures and colored by atom type. Spheres represent the pharmacophore features: red circles - negative ionic, blue sphere with arrows - hydrogen bonding donor, pink sphere with arrows - hydrogen bonding acceptor, green sphere - hydrophobic, and orange rings - aromatic features.
(TIFF)

S13 Fig Docking results of Ro318220 and ERK8 orthologues. (A) TbERK8 docking results with all Ro318220 poses generated and displayed by their stick structure and colored by energy scores, as in S10 Fig. (B) TbERK8 docking results with the best Ro318220 pose (#1) displayed as a stick structure and colored in yellow and by atom type, with Mg^2+^ as a green sphere. (C) HsERK8 Ro318220 docking results with all poses generated and displayed by their stick structure and colored by energy scores, as in S10 Fig. HsERK8 is shown as a navy cartoon with Mg^2+^ by a green sphere. (D) The HsERK8 docking with the best pose (#3) selected for interaction analysis. Ro318220 is displayed as a red stick structure, with an atom of Mg^2+^ as the green sphere.
(TIFF)

S14 Fig Docking results of AZ960 and ERK8 orthologues. (A) TbERK8 docking results with all poses displayed as stick structures and colored by energy scores, as in S10 Fig. TbERK8 is shown in violet cartoon with the Mg^2+^ ion as a green sphere. (B) TbERK8 docking results with best pose (#1) displayed by its stick structure and colored in red with the Mg^2+^ ion as a green sphere. (C) HsERK8 docking results with all poses displayed as stick structures and colored by energy scores, as in S10 Fig. HsERK8 is shown in navy cartoon with the Mg^2+^ ion as a green sphere. (D) HsERK8 docking results with best pose (#1) displayed as stick structures and colored in red and by atom type, with the Mg^2+^ ion as a green sphere.
(TIFF)

S15 Fig AZ960 aromatic interactions with ERK8 orthologues. (A) Full structure overlay of HsERK8 and TbERK8. (B) TbPhe156 interaction with AZ960. (C) HsPhe97 aromatic interaction with AZ960. (D) Overlay of Asp155 and Phe156 in HsERK8 and TbERK8, displaying a shift in residues. HsERK8 is shown by the navy cartoon, and interacting residues are shown as stick structures. TbERK8 is demonstrated in a violet cartoon, and interacting residues are shown as stick structures. AZ960 is displayed as stick structures, as shown in gray. The Mg^2+^ ion is shown as a green sphere.
(TIFF)

S16 Fig Docking results of famotidine and ERK8 orthologues. (A) TbERK8 docking results with all poses displayed as stick structures and colored by energy scores as in S10 Fig, with the Mg^2+^ ion as a green sphere. (B) HsERK8 docking results with all poses generated are displayed as stick structures and colored by energy scores, as in S10 Fig. HsERK8 is shown in navy cartoon with the Mg^2+^ ion as a green sphere. (C) TbERK8 docking results with best pose (#1) displayed as stick structures with red carbons, with the Mg^2+^ ion as a green sphere. (D) HsERK8 docking results with best pose (#1) displayed as stick structures with carbons colored in red and the Mg^2+^ ion as a green sphere.
(TIFF)

S17 Fig Docking results of fludrocortisone and ERK8 orthologues. (A) TbERK8 docking results with all poses generated are displayed as stick structures and colored by energy scores, as in S10 Fig. (B) HsERK8 docking results with all poses displayed as stick structures and colored as in S10 Fig. (C) TbERK8 docking results with best pose (#1) displayed as stick structures and colored as in S10 Fig. HsERK8 is shown in navy cartoon with Mg^2+^ as a green sphere. (D) HsERK8 docking results with best pose (#2) displayed as stick structures and carbons colored in orange and by atom type with the Mg^2+^ ion as a green sphere.
(TIFF)

S18 Fig Docking results of fluprednisolone and ERK8 orthologues. (A) TbERK8 docking results with all poses generated are displayed as stick structures and colored by energy scores as in S10 Fig. (B) HsERK8 docking results with all poses generated are displayed as stick structures and colored by energy scores, as in S10 Fig. (C) TbERK8 docking results with the best pose (#1) are displayed as stick structures, as in S10 Fig. HsERK8 is shown in navy cartoon in S10 Fig. (D) HsERK8 docking results with best pose (#1) displayed as stick structures and colored as in S10 Fig.
(TIFF)

S19 Fig Docking results of idarubicin and ERK8 orthologues. (A) TbERK8 docking results with all poses generated are displayed as stick structures and colored as in S10 Fig. (B) HsERK8 docking results with all poses generated are displayed as stick structures and colored as in S10 Fig. (C) TbERK8 docking results with best pose (#1) displayed by its stick structure and colored as in S10 Fig. HsERK8 is shown as navy and colored, as shown in S10 Fig. (D) HsERK8 docking results with best pose (#1) displayed by its stick structure and colored as in S10 Fig.
(TIFF)

S20 Fig Docking results of prednisolone and ERK8 orthologues. (A) TbERK8 docking results with all poses displayed as stick structures and colored as in S10 Fig. (B) HsERK8 docking results with all poses displayed as stick structures and colored as in S10 Fig. HsERK8 is shown as a navy cartoon and colored as in S10 Fig. (C) TbERK8 docking results with best pose (#2) displayed by its stick structure and colored as in S10 Fig. (D) HsERK8 docking results with the best pose (#2) displayed by its stick structure and colored as in S10 Fig.
(TIFF)

S21 Fig Docking results of sildenafil and ERK8 orthologues. (A) TbERK8 docking results with all poses displayed as stick structures and colored as in S10 Fig. (B) HsERK8 docking results with all poses displayed as stick structures and colored as in S10 Fig. HsERK8 is shown as a navy cartoon and colored as in S10 Fig. (C) TbERK8 docking results with the best pose (#1) displayed by its stick structure and colored as in S10 Fig. (D) HsERK8 docking results with the best pose (#1) displayed by its stick structure and colored as in S10 Fig.
(TIFF)

S22 Fig Prednisolone and Sildenafil dose curves done with recombinant HsERK8 and TbERK8. (A) Purification of GST-HsERK8 by GST-agarose column chromatography after overexpression in *E. coli*. Arrow points to eluted fractions containing ~84 kDa GST-HsERK8 fusion protein stained in SDSPAGE with Bio-Rad QC Colloidal Coomassie G-250. (B) Purification of GST-Tb ERK8 by GST-agarose column chromatography after overexpression in *E. coli*. Arrow points to eluted fractions containing ~74 kDa GST-TbERK8 fusion protein stained in SDSPAGE with Coomassie Brilliant Blue R-250 (For SDSPAGE, crude lysate [C], flow through [FT], wash [W], numbered lanes are eluted fractions). (C) Dose curves for kinase assays comparing prednisolone inhibition of HsERK8 and TbERK8. (D) Dose curves for kinase assays comparing Sildenafil inhibition of HsERK8 and TbERK8. Curves were generated by Prism 8.1.1 (GraphPad) from kinase assays testing 4-fold serial dilution of each drug at concentrations ranging from 10 mM to 150 nM for their inhibitor potency. Each point in the dose curve represents the mean value with standard deviation from three independent assays done on different days.
(TIFF)

S23 Fig Ligand efficiencies of known inhibitors and FDA-approved molecules. Ligand efficiencies are calculated in kcal/mol/heavy atom count. (A) TbERK8 ligand efficiencies are organized from highest efficiency to lowest. (B) HsERK8 ligand efficiencies are organized from highest efficiency to lowest. (C) Comparison of TbERK8 and HsERK8 ligand efficiencies organized by size (Å^3^) of each ligand. Ligand efficiency calculated using all poses output energies from AutoDock Vina and heavy atom numbers and standard deviations.
(TIFF)

S24 Fig Ligand binding energies and efficiencies of known inhibitors and constructed chemical compounds. (A) Ligand efficiency was calculated using all pose output energies from AutoDock Vina. (B) Binding energies were evaluated using all pose outputs from AutoDock Vina and Schrödinger-Maestro. The molecular mechanics/generalized Born surface area (MM-GBSA) feature was used to calculate the energy of complexes and standard deviations.
(TIFF)

S1 Raw Data Excel.xlsx spreadsheet file with the raw Relative Luminescence Unit (RLU) values for the ATP-bioluminescence assay measured by luminometer. (XLSX)

S1 Table Summary table of sequence identity and similarity between Fus3 (PDB ID: 2b9f), TbERK8, HsERK8, and the MAPK template (PDB ID: 3oz6) sequences. Sequence identity and similarity percentages were calculated using Schrödinger-Maestro after performing pairwise sequence alignment. Percent similarity was calculated using the BLOSUM62 matrix.
(TIFF)

S2 Table The key residue names and sequence positions of Fus3, TbERK8, and HsERK8 were based on active site overlay and literature. Residues are renumbered to maintain consistency in the discussion of amino acid position based on sequence, and renumbered residues are based on the sequence position in HsERK8.
(TIFF)

S3 Table Docking free energies of poses for ADP redocked into the Fus3 energy-minimized structure. Energies are shown in kcal/mol. AutoDock Vina was used for docking, which generates up to nine poses. The pose used in the analysis was determined by the lowest energy score and frequency of additional poses with similar positioning, correct positioning of chemical groups, and similarity to ADP in the crystal structure (represented by an asterisk).
(TIFF)

S4 Table Docking free energies of poses for ATP docked into Fus3, TbERK8, and HsERK8 energy-minimized structures. Energies are shown in kcal/mol. AutoDock Vina was used for docking, which generates up to nine poses. The pose used in the analysis was determined from the lowest energy score and frequency of additional poses with similar positioning, the correct positioning of chemical groups, and similarity to ADP in the crystal structure (represented by an asterisk).
(TIFF)

S5 Table Small Molecules used for docking into ERK8 orthologues. (TIFF)

S6 Table Docking free energies of poses for Ro318220 docked into the TbERK8 and HsERK8 energy-minimized structure. Energies are shown in kcal/mol. AutoDock Vina was used for docking, which generates up to nine poses. The pose used in the analysis was determined from the lowest energy score and the frequency of additional poses with similar positioning (represented by an asterisk).
(TIFF)

S7 Table Docking free energies of poses for AZ960 docked into the TbERK8 and HsERK8 energy-minimized structures. Energies are shown in kcal/mol. AutoDock Vina was used for docking, which generates up to nine poses. The pose used in the analysis was determined by the lowest energy score and frequency of additional poses with similar positioning (represented by an asterisk).
(TIFF)

S8 Table Protein-ligand interactions of analyzed inhibitors with TbERK8 and HsERK8 from the molecular docking results. Interactions were identified using the Schrödinger-Maestro fingerprinting feature. Distances less than 5 Å are given in parenthesis (in Ångstroms, from heavy atom to heavy atom, determined using the best ligand poses visualized in PyMol). Electrostatic distances were calculated from the nearest charged protein atom to the nearest polar ligand atom (including cyano groups). Hydrophilic distances were calculated from the nearest polar protein atom to the nearest polar ligand atom. Hydrophobic distances were calculated from the nearest non-polar protein atom to the nearest non-polar ligand atom. Backbone distances were calculated from the nearest backbone protein atom to the ligand atom. “Difference” indicates the net number of residues involved in an interaction of that type with the parasite ortholog versus the human.
(TIFF)

S9 Table Docking free energies of poses for famotidine docked into the TbERK8 and HsERK8 energy-minimized structures. Energies are shown in kcal/mol. AutoDock Vina was used for docking, which generates up to nine poses. The pose used in the analysis was determined by the lowest energy score and frequency of additional poses with similar positioning (represented by an asterisk).
(TIFF)

S10 Table Docking free energies of poses for fludrocortisone docked into the TbERK8 and HsERK8 energy minimized structures. Energies are shown in kcal/mol. AutoDock Vina was used for docking, which generates up to nine poses. The pose used in the analysis was determined by the lowest energy score and frequency of additional poses with similar positioning (represented by an asterisk).
(TIFF)

S11 Table Docking free energies of poses for fluprednisolone docked into the TbERK8 and HsERK8 energy-minimized structures. Energies are shown in kcal/mol. AutoDock Vina was used for docking, which generates up to nine poses. The pose used in the analysis was determined by the lowest energy score and the frequency of additional poses with similar positioning (represented by an asterisk).
(TIFF)

S12 Table Docking free energies of poses for idarubicin docked into the TbERK8 and HsERK8 energy-minimized structures. Energies are shown in kcal/mol. AutoDock Vina was used for docking, which generates up to nine poses. The pose used in the analysis was determined by the lowest energy score and the frequency of additional poses with similar positioning (represented by an asterisk).
(TIFF)

S13 Table Docking free energies of poses for prednisolone docked into the TbERK8 and HsERK8 energy-minimized structures. Energies are shown in kcal/mol. AutoDock Vina was used for docking, which generates up to nine poses. The pose used in the analysis was determined by the lowest energy score and the frequency of additional poses with similar positioning (represented by an asterisk).
(TIFF)

S14 Table Docking free energies of poses for sildenafil docked into the TbERK8 and HsERK8 energy-minimized structures. Energies are shown in kcal/mol. AutoDock Vina was used for docking, which generates up to nine poses. The pose used in the analysis was determined by the lowest energy score and the frequency of additional poses with similar positioning (represented by an asterisk).
(TIFF)

S15 Table Protein-small molecule interactions of identified FDA-approved drugs with similar physicochemical characteristics to Ro318220 and AZ960 with TbERK8 and HsERK8 from molecular docking results. Interactions were determined using the Schrödinger-Maestro fingerprinting feature.
(TIFF)

S16 Table New compound constructs built from the AZ960 scaffold, number of rotatable bonds, heavy atoms, and volumes. (TIFF)

S17 Table Docking free energies of new small molecule constructs docked into the HsERK8 energy-minimized structure. Energies are shown in kcal/mol. AutoDock Vina was used for docking, which generates up to nine poses. The pose used in the analysis was determined by the lowest energy score and the frequency of additional poses with similar positioning (represented by an asterisk).
(TIFF)

S18 Table Docking free energies of new small molecule constructs docked into the TbERK8 energy-minimized structure. Energies are shown in kcal/mol. AutoDock Vina was used for docking, which generates up to nine poses. The pose used in the analysis was determined by the lowest energy score and the frequency of additional poses with similar positioning (represented by an asterisk).
(TIFF)

S19 Table Protein-small molecule interactions of new chemical compounds with TbERK8 and HsERK8 from molecular docking results. Interactions were identified using the Schrödinger-Maestro fingerprinting feature.
(TIFF)

S1 File Experimental Methods. (DOCX)
