# Supplementary figures and images for: Molecular docking to homology models of human and Trypanosoma brucei ERK8 that identified ortholog-specific inhibitors
Source: PLoS Negl Trop Dis. 2025 Sep 12;19(9):e0013487. doi: 10.1371/journal.pntd.0013487 (PMC12445744; doi:10.1371/journal.pntd.0013487)

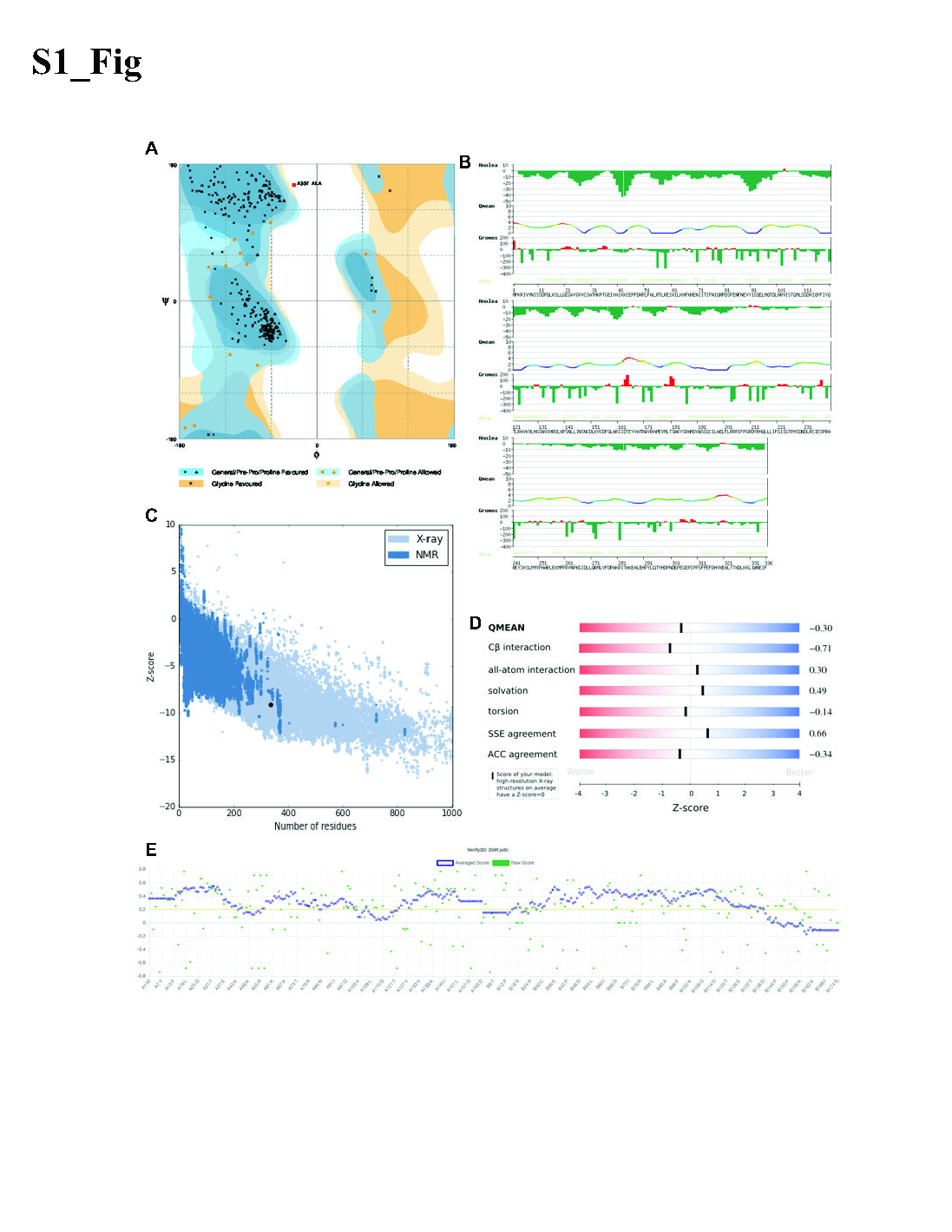

Supplement: S1 Fig — (A) Ramachandran plot showed 95.5% of residues within the favored region, 4.2% within the allowed region, and 0.3% in the outlier region. (B) ANOLEA indicates favorable free energy scores (green). No major unfavorable residue regions were observed. (C) ProSA indicates a Z-score (-9.11, black dot) in the acceptable spectrum compared to resolved protein structures. (D) QMEAN analysis indicates mostly favorable Z-scores in solvation, SSE agreement, and all-atom interactions. Negative Z-scores were observed for torsion, ACC agreement, and CB interactions. (E) Verify3D results showed most residues above the threshold for favorable side chain placement (dotted yellow line). Residue ranges below the threshold are not within the active site. (TIFF) [file pntd.0013487.s001.tiff]

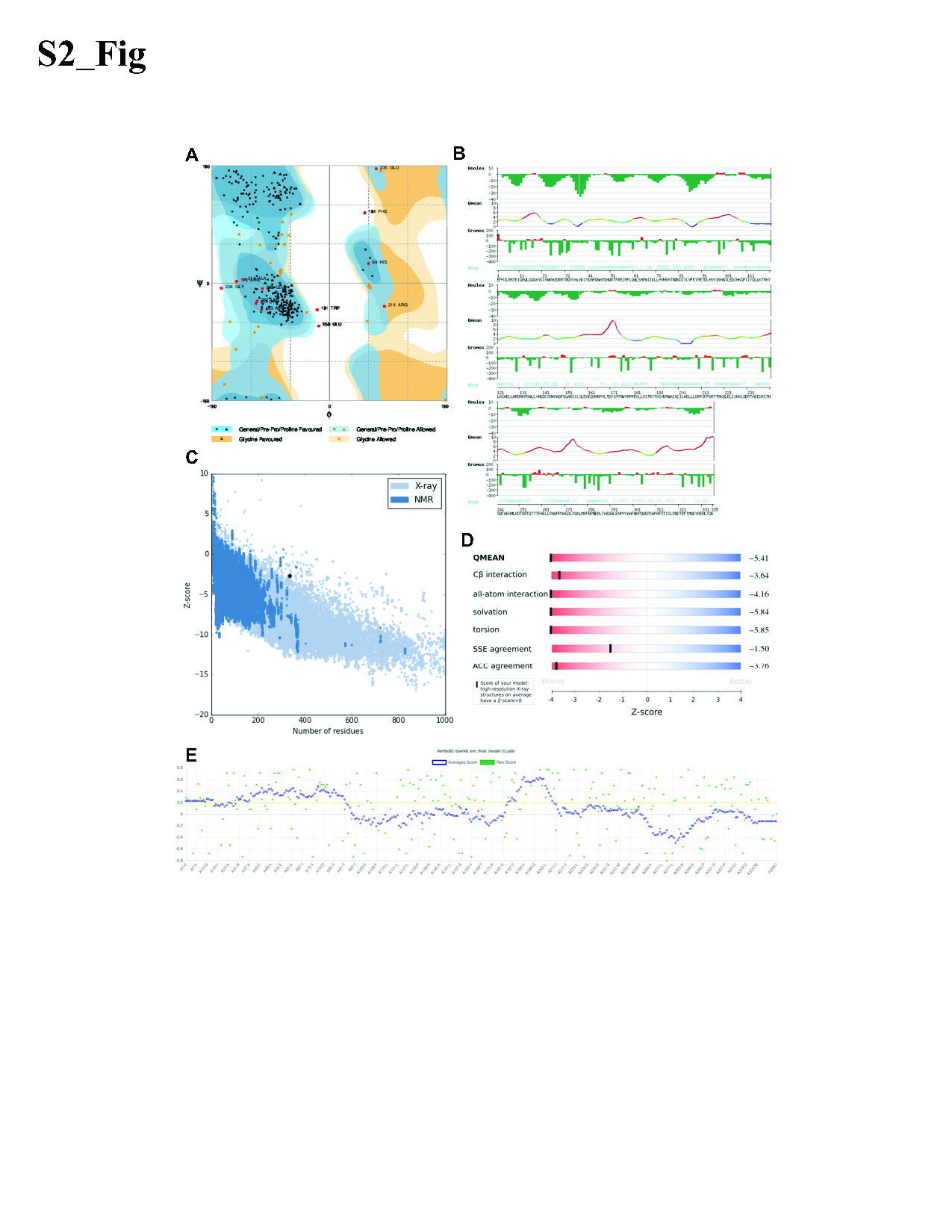

Supplement: S2 Fig — (A) Ramachandran plot showed 88% of residues within the favored region, 8.1% within the allowed region, and 3.9% in the outlier region. (B) ANOLEA indicates favorable free energy scores (green). No major unfavorable residue regions were observed. (C) ProSA indicates a Z-score (-2.7, black dot) in the acceptable spectrum compared to resolved protein structures. (D) QMEAN analysis indicated negative Z-scores in all analysis areas. (E) Verify3D results showed most residues above the threshold for favorable side chain placement (dotted yellow line). Residue ranges below the threshold are not within the active site. (TIFF) [file pntd.0013487.s002.tiff]

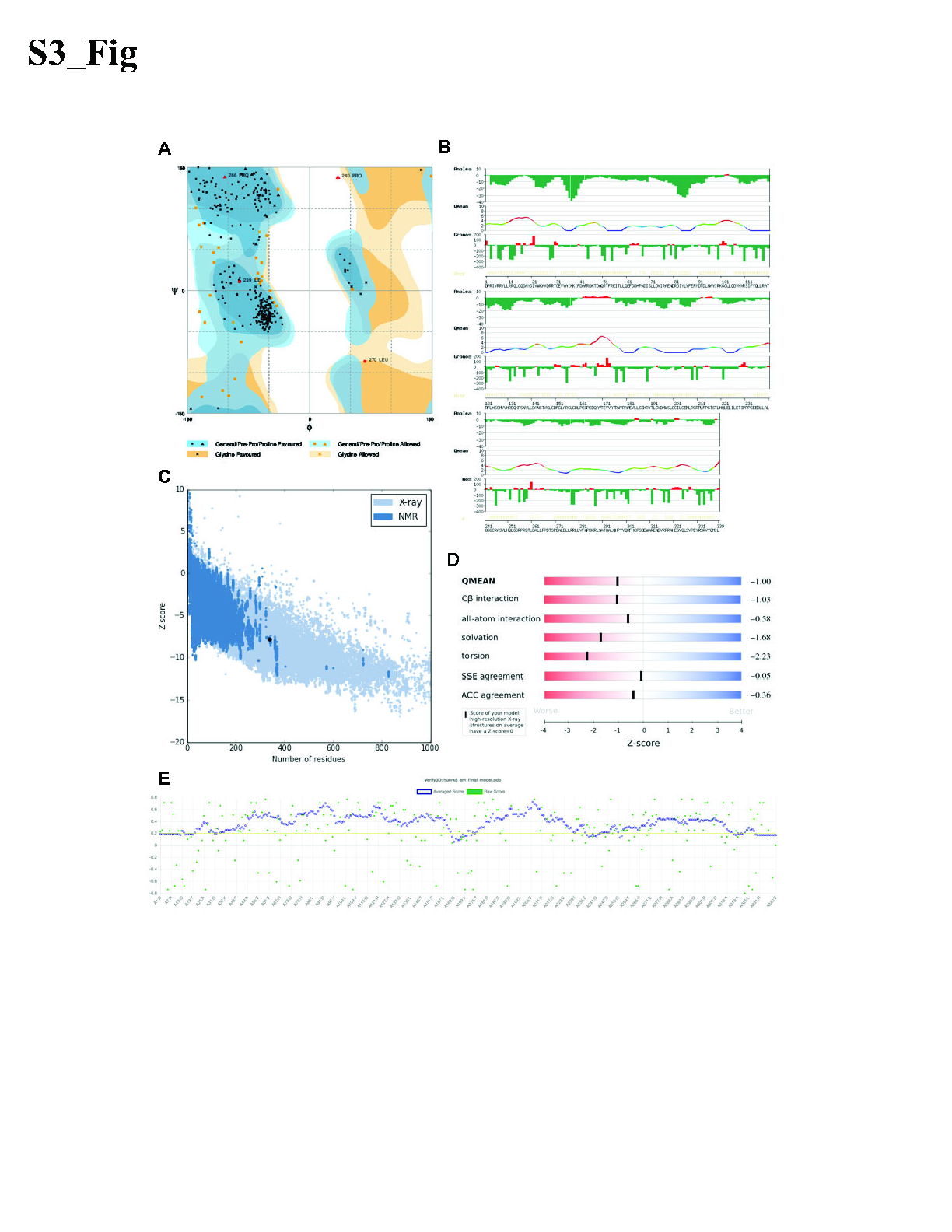

Supplement: S3 Fig — (A) Ramachandran plot showed 89.3% of residues within the favored region, 9.5% within the allowed region, and 1.2% in the outlier region. (B) ANOLEA indicates favorable free energy scores (green). No major unfavorable residue regions were observed. (C) ProSA indicates a Z-score (-7.87, black dot) in the acceptable spectrum compared to resolved protein structures. (D) QMEAN analysis indicated negative Z-scores in all analysis areas. (E) Verify3D results showed most residues above the threshold for favorable side chain placement (dotted yellow line). Residue ranges below the threshold are not within the active site. (TIFF) [file pntd.0013487.s003.tiff]

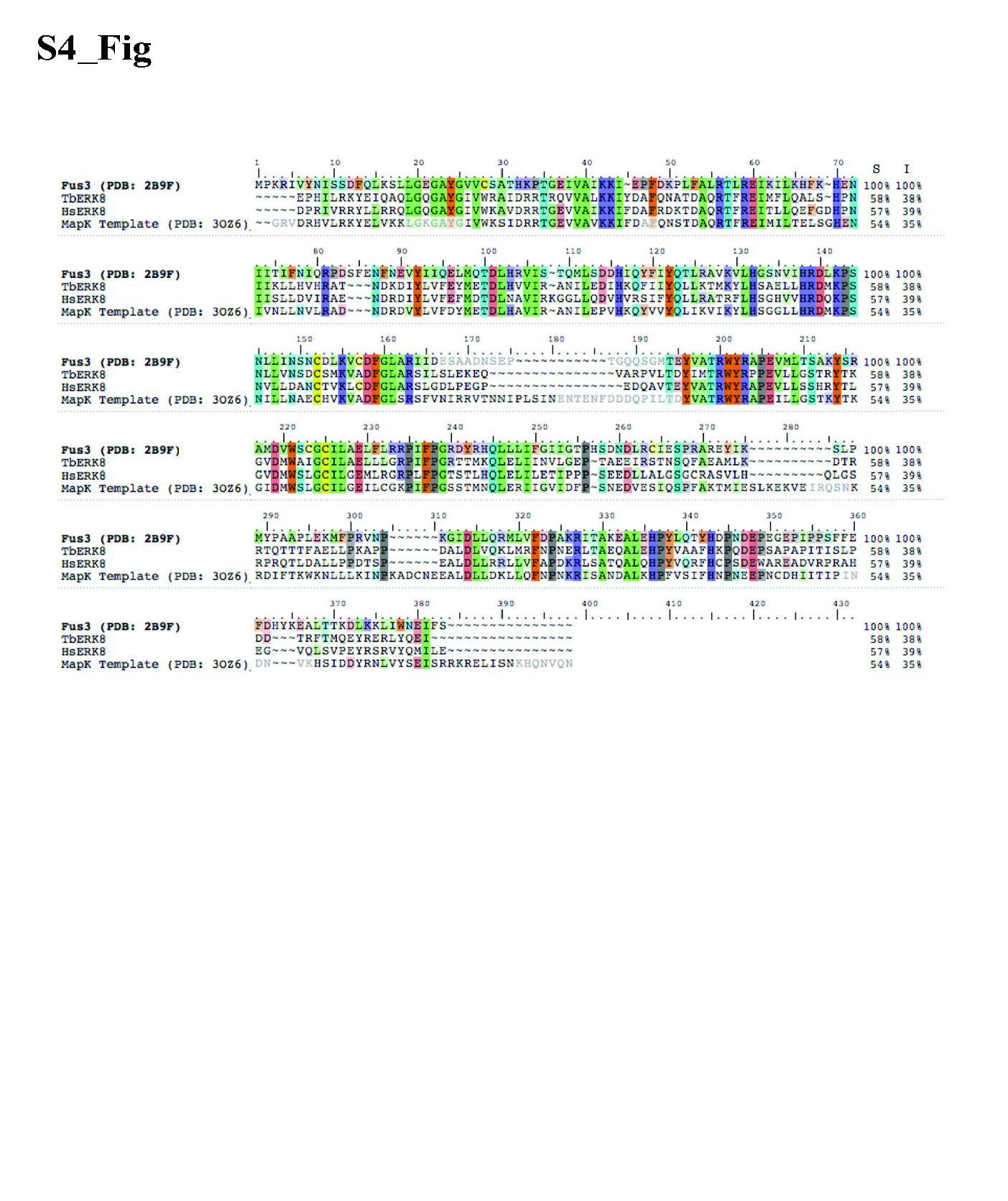

Supplement: S4 Fig — Alignment performed using Schrödinger-Maestro’s multiple sequence viewer. Sequences are displayed as single-letter amino acids, with conserved residues colored based on side-chain property and alignment quality. The Fus3 sequence was used as the reference, with percent similarity (S) and identity (I) indicated in the right column. (TIFF) [file pntd.0013487.s004.tiff]

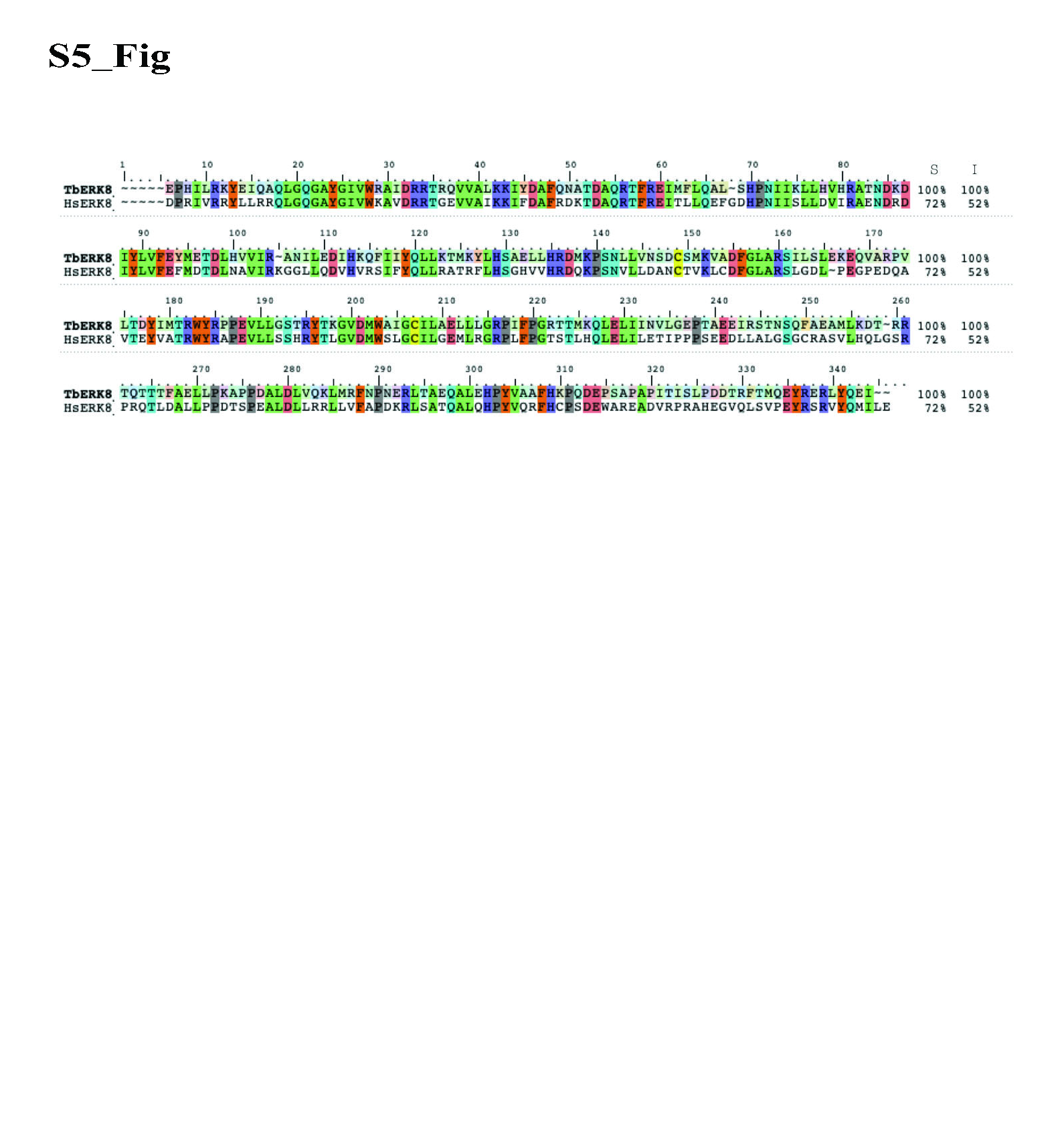

Supplement: S5 Fig — Alignment performed using Schrödinger-Maestro’s multiple sequence viewer. Sequences are displayed as single-letter amino acids, with conserved residues colored based on side-chain property and alignment quality. The right column indicates percentage similarity (S) and identity (I). (TIFF) [file pntd.0013487.s005.tiff]

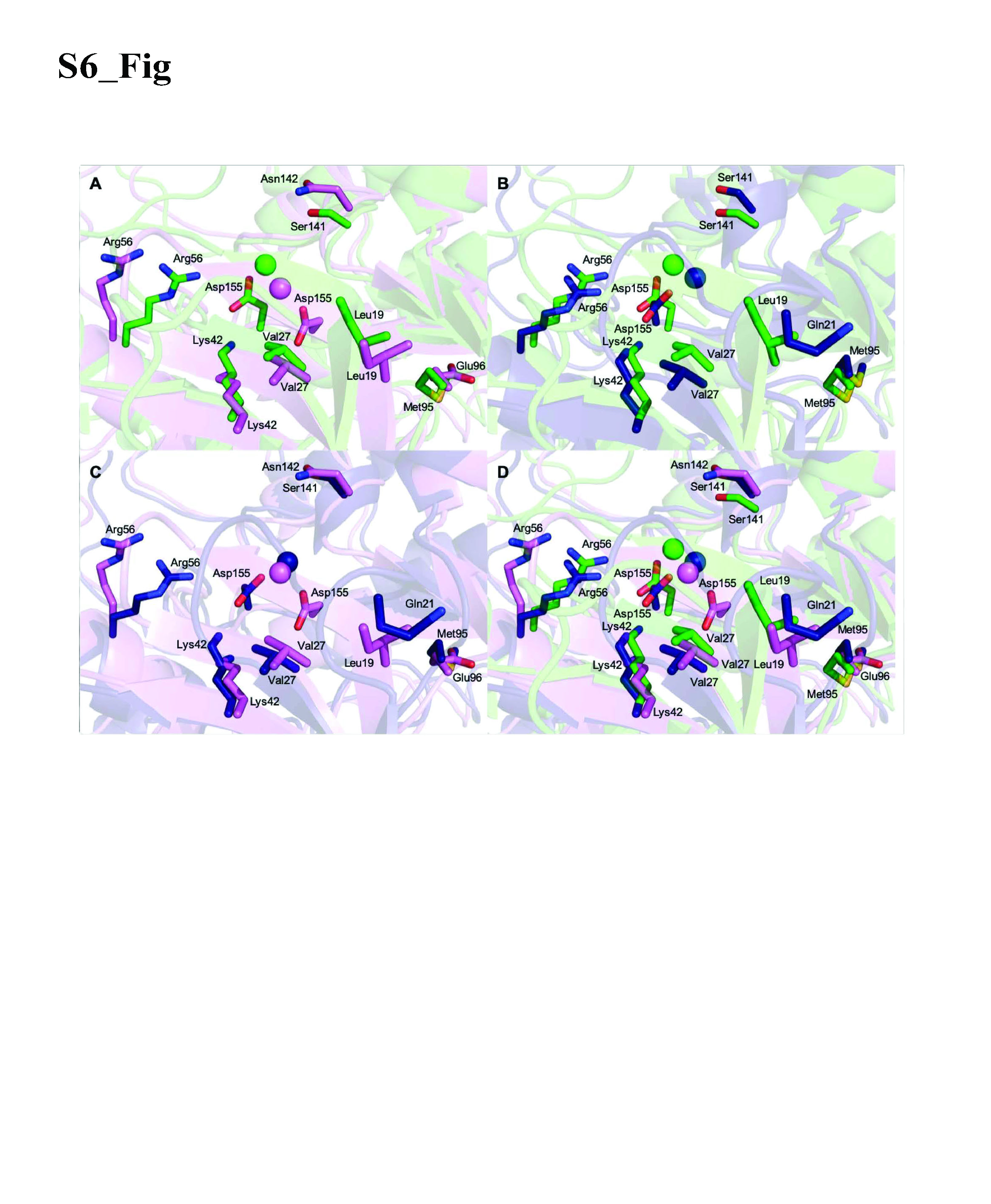

Supplement: S6 Fig — (A) Fus3 and TbERK8, (B) Fus3 and HsERK8, (C) HsERK8 and TbERK8, and (D) Fus3, TbERK8, and HsERK8. All structures are shown as cartoons and colored as Fus3 (green), TbERK8 (violet), and HsERK8 (navy). Residues in the ATP binding cavity are labeled and shown as stick structures and colored based on the kinase orthologue. Mg2+ is shown as spheres and colored based on the kinase orthologue. Sphere scale set to 0.6 Å. (TIFF) [file pntd.0013487.s006.tiff]

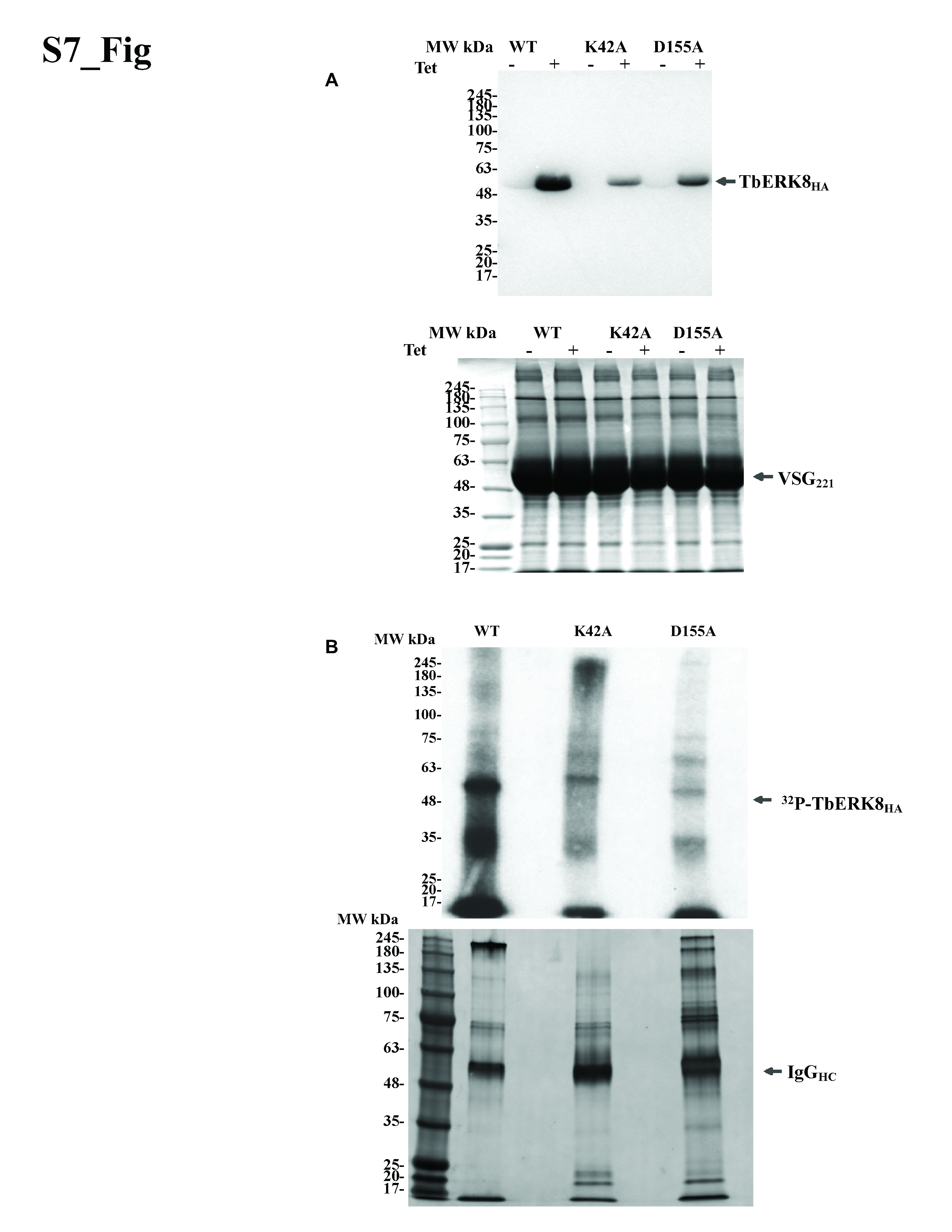

Supplement: S7 Fig — Expression of TbERK8HA constructs in the bloodstream form of T. brucei. Extracts isolated from control or tetracycline-induced T. brucei expressing wild-type TbERK8HA were grown in a culture medium for 24 h and then prepared for examination. (A) Immunoblot assay against lysates from control (Tet-) or induced (Tet+) T. brucei strains that overexpress TbERK8HA (Top Panel. Arrow points to 52kDa TbERK8HA band recognized by α-HA). Each lane represents lysates from about 2.5 x10^6^ parasites for immunoblot loading control (Bottom Panel. Arrow points to ~55kDa VSG221 band in corresponding Coomassie-stained gel in loading control). (B) Immuno-precipitation (IP) and 32P-autoradiography of TbERK8 variants. Lysates from 108 tetracycline-induced T. brucei strains, each overproducing TbERK8-WTHA, TbERK8-K42AHA, or TbERK8-D155AHA variants, were incubated with anti-HA antibodies for 24 h. Each lysate was incubated with Protein A agarose beads overnight and washed. The beads were incubated with kinase buffer containing 10μCi 32P-γ- ATP, washed, resolved by SDS-PAGE, and examined by autoradiography (Top Panel. Arrow points to the 52kDa auto-phosphorylated TbERK8HA band. Variability of band size in the D155A lane may result from the reduced intensity of labeling by 32P-γ-ATP, indicative of its decrease in auto-phosphorylation. The variability of band size in the K42A lane likely results from kinase activity unrelated to TbERK8 captured by the IP beads, as previously observed [19]). (Bottom panel. Silver-stained SDSPAGE loading control from 32P-IP assay. Arrow points to ~55kDa immunoglobulin G heavy chain (IgGHC)) Ladder shown is GoldBio Bluestain. (TIFF) [file pntd.0013487.s007.tiff]

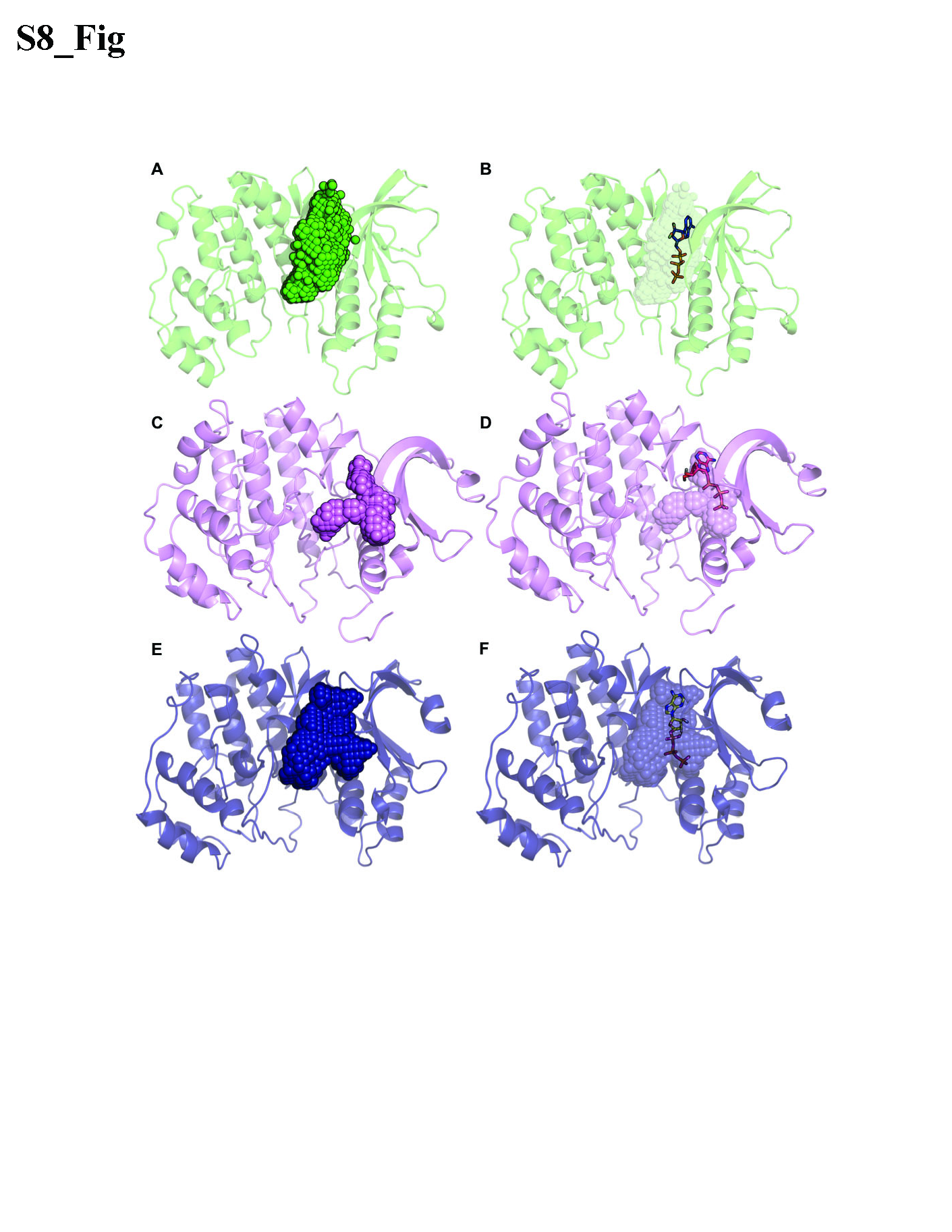

Supplement: S8 Fig — The volume pocket of the ATP binding cavity was created using MetaPocket 2.0 and calculated with UCSF Chimera. (A, C, E) The binding pocket is shown as spheres to indicate the volume and shape of the ATP binding cavity for each structure. (B, D, F) Overlays of the volume representation with ATP were also performed to highlight the orientation of ATP in the predicted pocket. All structures are shown as cartoons and colored by Fus3 (green), TbERK8 (violet), and HsERK8 (navy). ATP is shown by its stick structure and colored by element. (TIFF) [file pntd.0013487.s008.tiff]

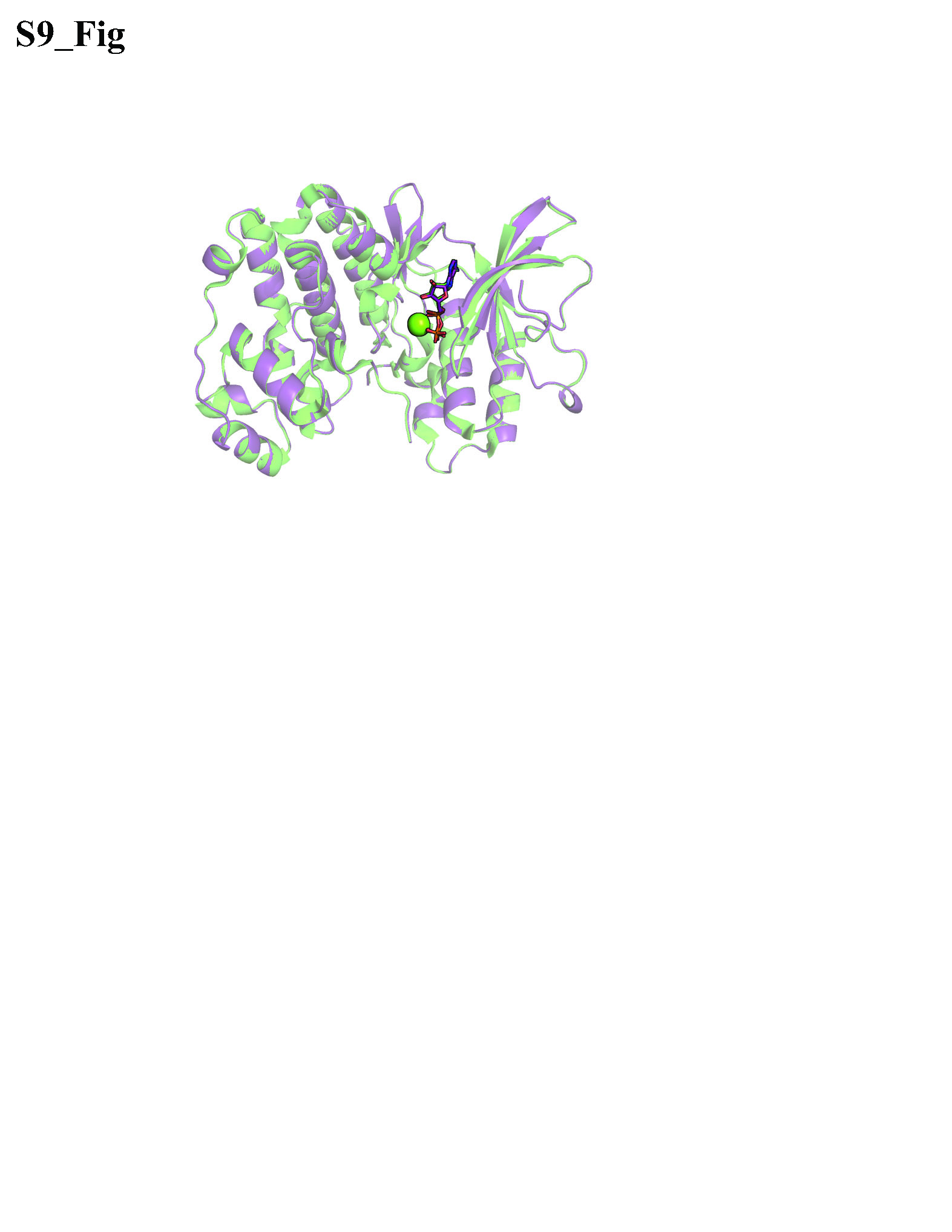

Supplement: S9 Fig — The Fus3 structure is shown in a cartoon and colored as an X-ray crystal structure (green), and the energy-minimized structure is used for redocking (purple). The lowest energy docked pose of ADP docking results (pose 1) is overlaid with the crystal structure position of ADP. ADP is shown by its stick structure and colored by structure (X-ray crystal structure in green, re-docked pose in purple). The docked pose had an RMSD of 0.710 Å compared to the X-ray crystal structure pose. (TIFF) [file pntd.0013487.s009.tiff]

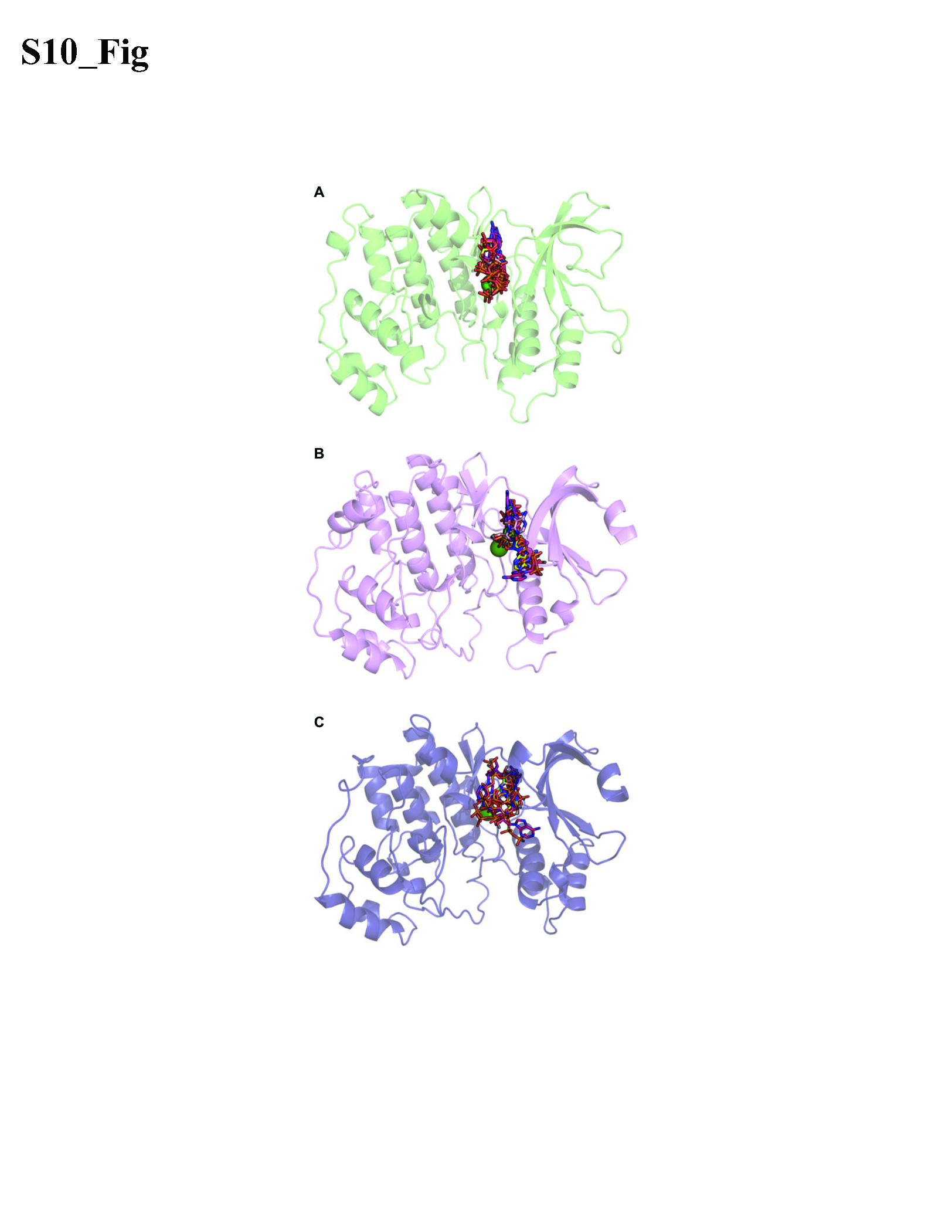

Supplement: S10 Fig — (A) Fus3 docking results with all poses displayed as stick structures and colored by energy scores in red, orange, yellow, green, blue, and violet (ROYGBV). Fus3 is shown as a green cartoon with Mg2+ as a green sphere. (B) TbERK8 docking results with all poses displayed as stick structures and colored by energy scores in ROYGBV. TbERK8 is shown in violet cartoon with Mg2+ in green sphere. (C) HsERK8 docking results with all poses generated are displayed as stick structures and colored by energy scores in ROYGBV. HsERK8 is shown in navy cartoon with Mg2+ in green sphere. (TIFF) [file pntd.0013487.s010.tiff]

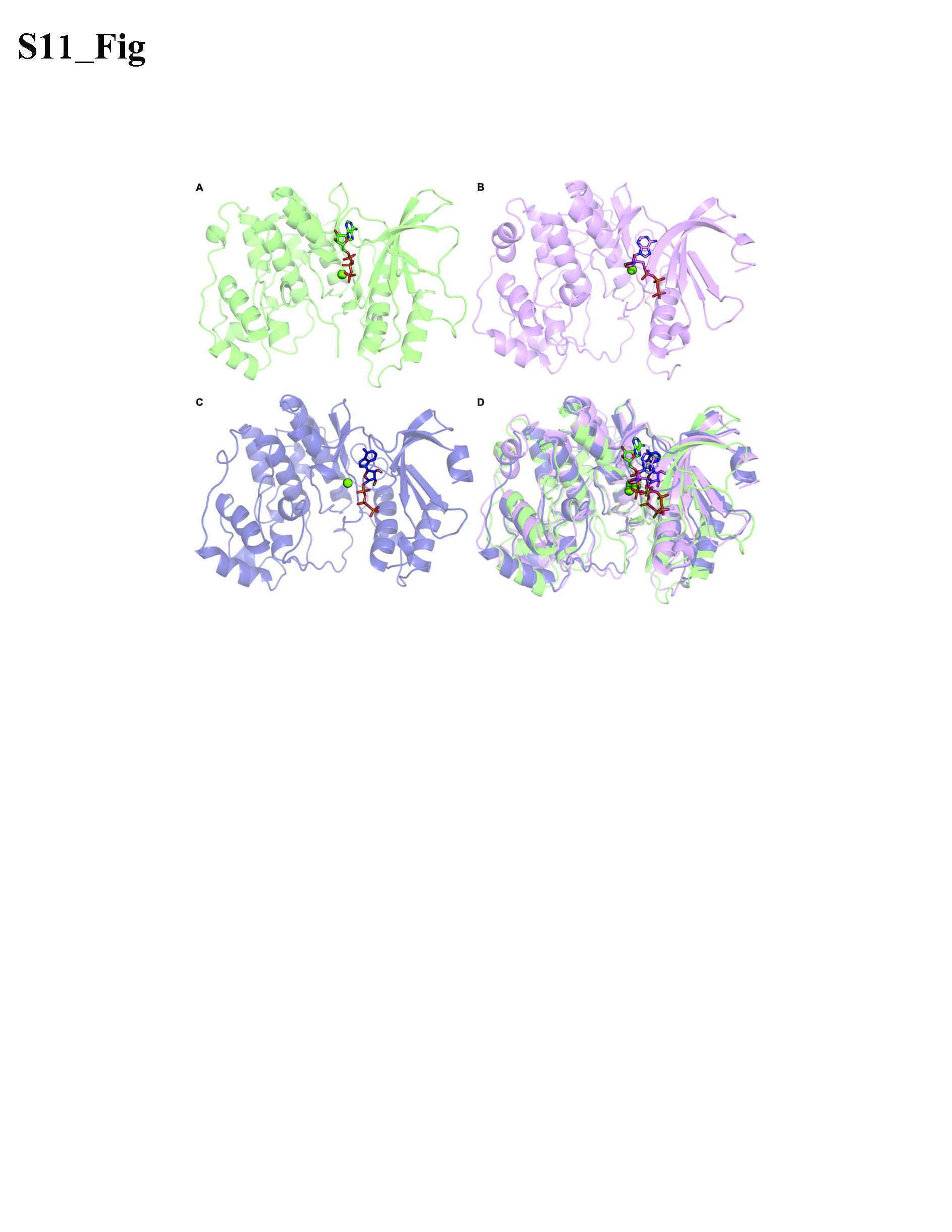

Supplement: S11 Fig — (A) Fus3 with best pose (#1) of ATP docking results, (B) TbERK8 with best pose (#1) of ATP, (C) HsERK8 with best pose (#3) of ATP, and (D) all three structures overlaid. Fus3, TbERK8, and HsERK8 are shown in cartoon colored green, violet, and navy, respectively, with Mg2+ in green sphere. ATP structures are represented by their stick structure and colored by associated protein and atom type. RMSD of ATP sans γ phosphate pose compared to ADP in the Fus3 crystal structure is 2.028 Å, 1.811 Å, and 1.920 Å, respectively. (TIFF) [file pntd.0013487.s011.tiff]

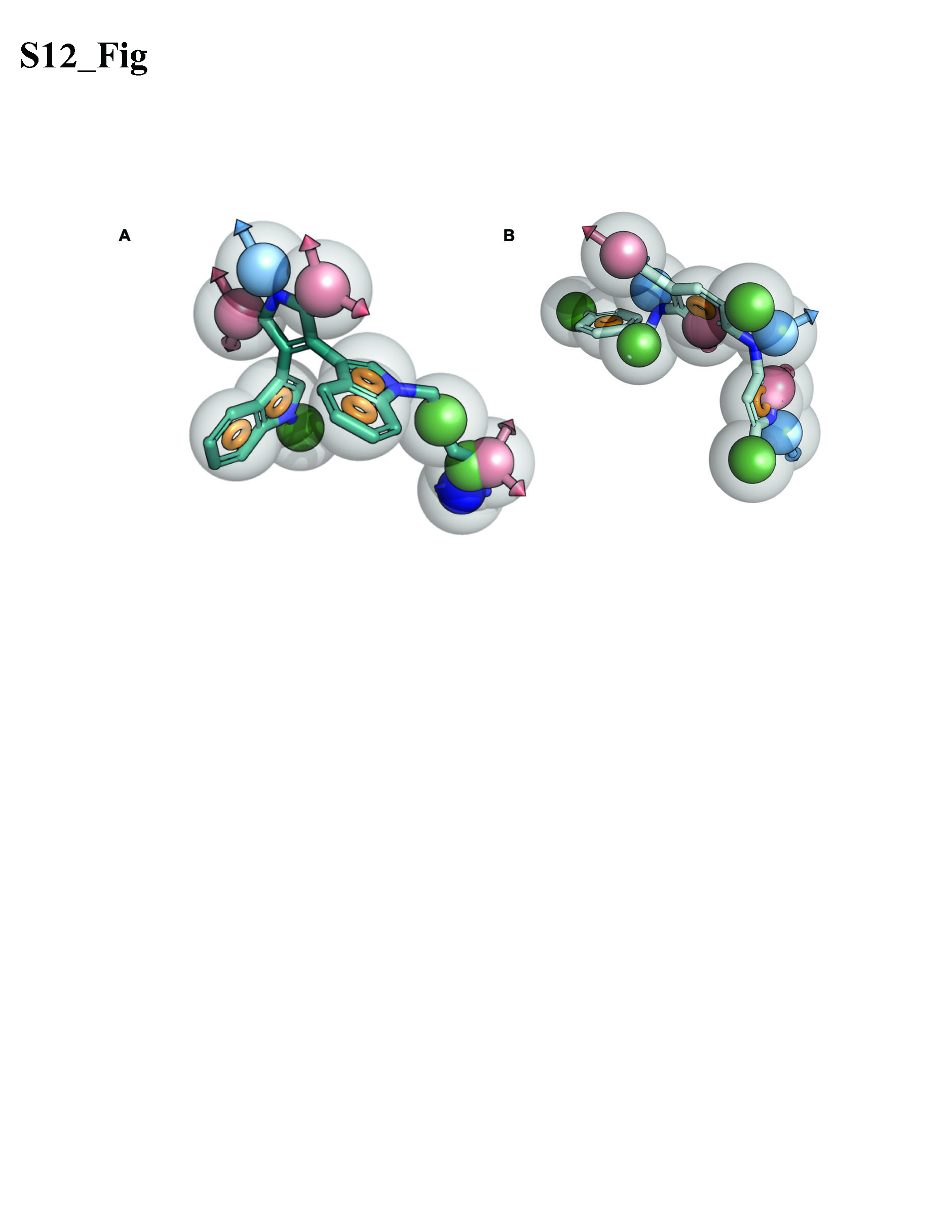

Supplement: S12 Fig — (A) Ro318220 (teal) and (B) AZ960 (light blue) are displayed as stick structures and colored by atom type. Spheres represent the pharmacophore features: red circles - negative ionic, blue sphere with arrows - hydrogen bonding donor, pink sphere with arrows - hydrogen bonding acceptor, green sphere - hydrophobic, and orange rings - aromatic features. (TIFF) [file pntd.0013487.s012.tiff]

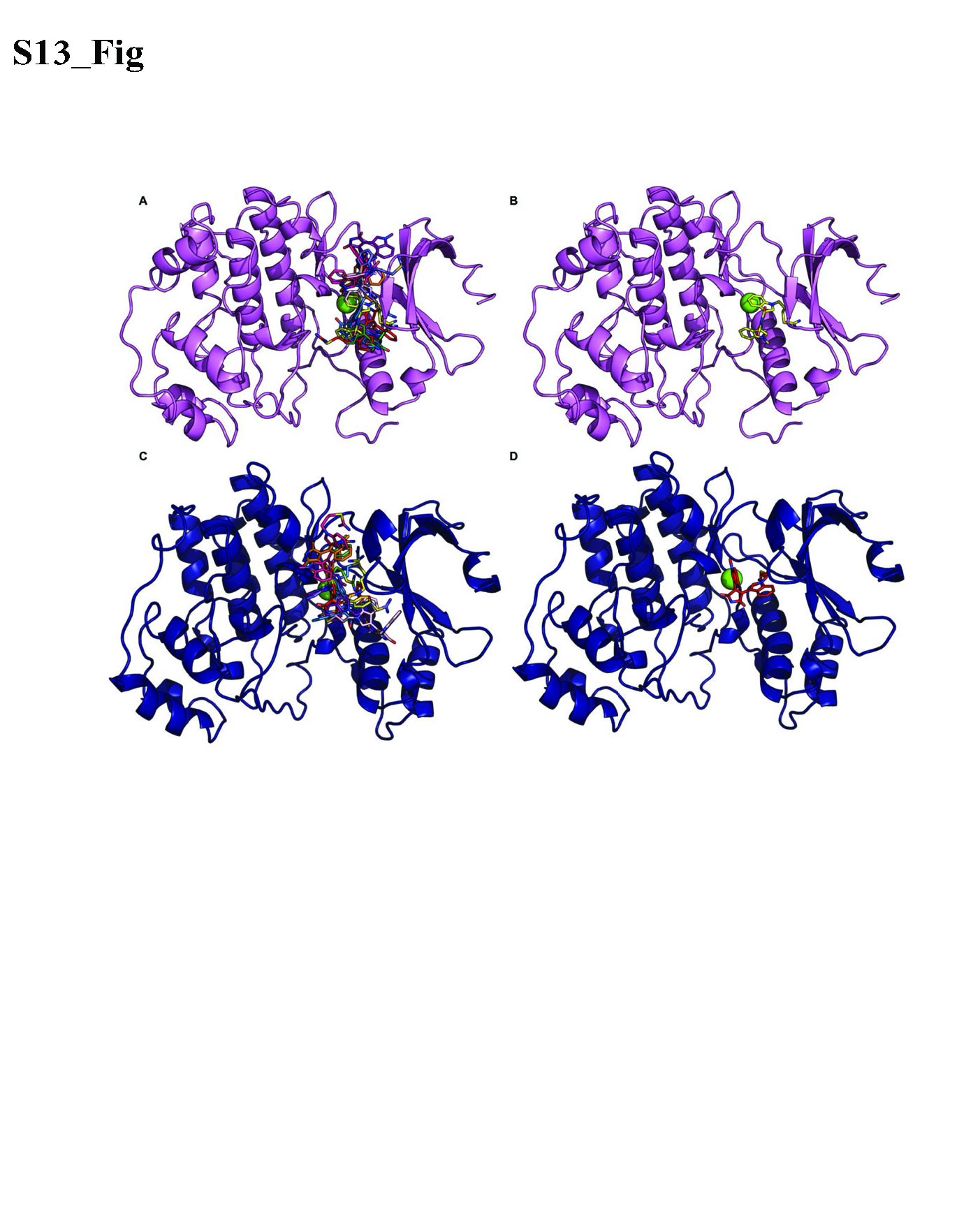

Supplement: S13 Fig — (A) TbERK8 docking results with all Ro318220 poses generated and displayed by their stick structure and colored by energy scores, as in S10 Fig. (B) TbERK8 docking results with the best Ro318220 pose (#1) displayed as a stick structure and colored in yellow and by atom type, with Mg2+ as a green sphere. (C) HsERK8 Ro318220 docking results with all poses generated and displayed by their stick structure and colored by energy scores, as in S10 Fig. HsERK8 is shown as a navy cartoon with Mg2+ by a green sphere. (D) The HsERK8 docking with the best pose (#3) selected for interaction analysis. Ro318220 is displayed as a red stick structure, with an atom of Mg2+ as the green sphere. (TIFF) [file pntd.0013487.s013.tiff]

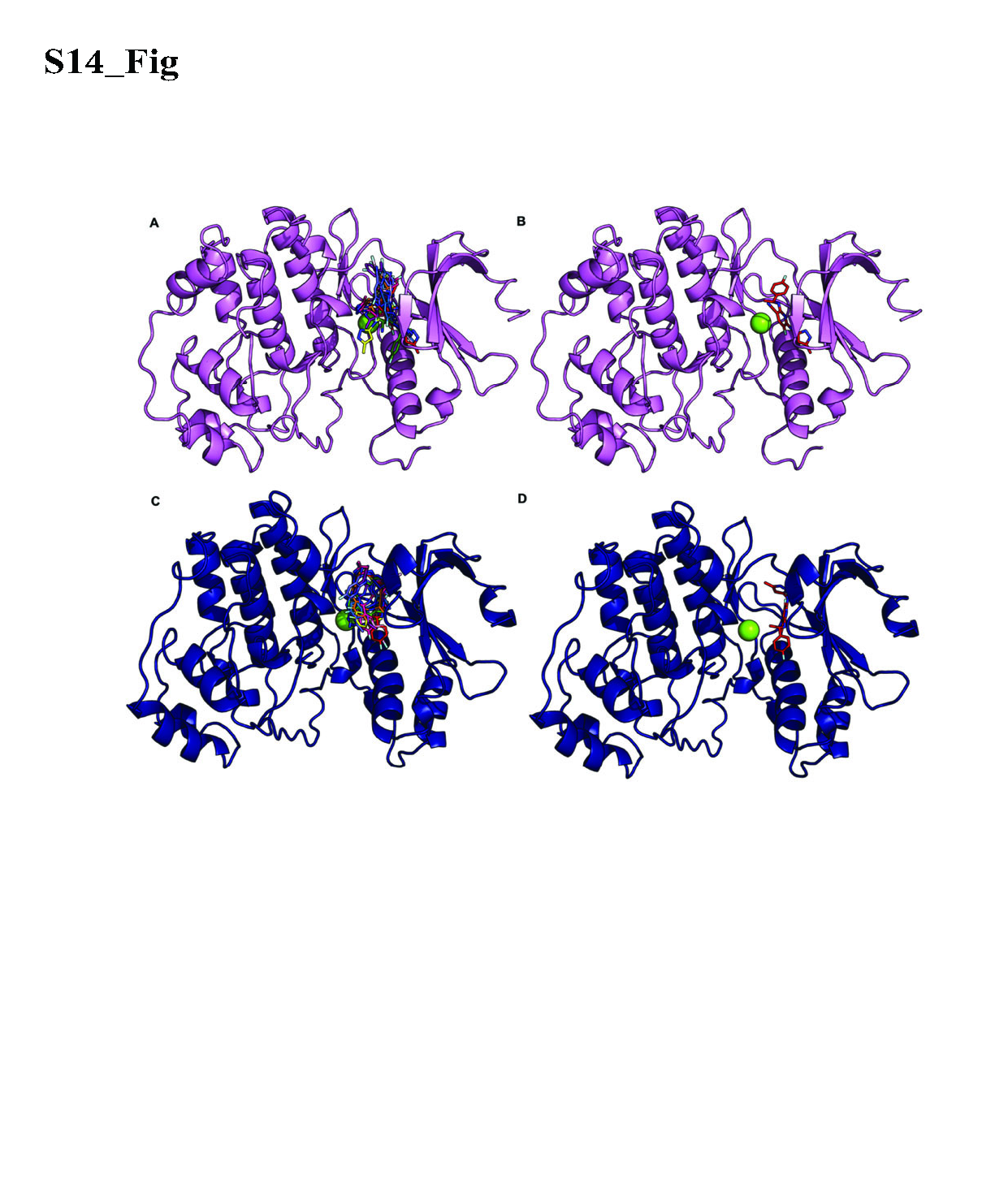

Supplement: S14 Fig — (A) TbERK8 docking results with all poses displayed as stick structures and colored by energy scores, as in S10 Fig. TbERK8 is shown in violet cartoon with the Mg2+ ion as a green sphere. (B) TbERK8 docking results with best pose (#1) displayed by its stick structure and colored in red with the Mg2+ ion as a green sphere. (C) HsERK8 docking results with all poses displayed as stick structures and colored by energy scores, as in S10 Fig. HsERK8 is shown in navy cartoon with the Mg2+ ion as a green sphere. (D) HsERK8 docking results with best pose (#1) displayed as stick structures and colored in red and by atom type, with the Mg2+ ion as a green sphere. (TIFF) [file pntd.0013487.s014.tiff]

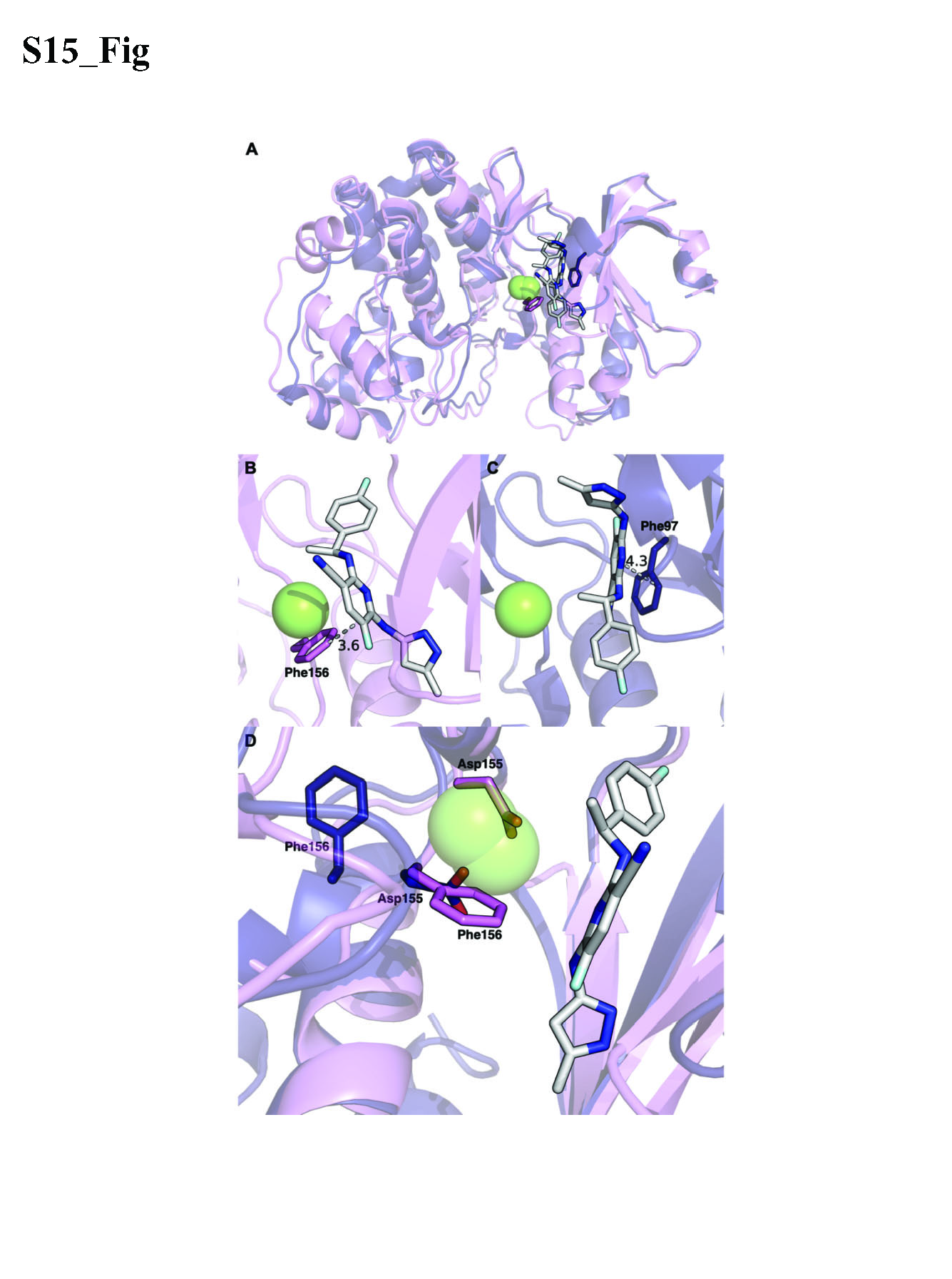

Supplement: S15 Fig — (A) Full structure overlay of HsERK8 and TbERK8. (B) TbPhe156 interaction with AZ960. (C) HsPhe97 aromatic interaction with AZ960. (D) Overlay of Asp155 and Phe156 in HsERK8 and TbERK8, displaying a shift in residues. HsERK8 is shown by the navy cartoon, and interacting residues are shown as stick structures. TbERK8 is demonstrated in a violet cartoon, and interacting residues are shown as stick structures. AZ960 is displayed as stick structures, as shown in gray. The Mg2+ ion is shown as a green sphere. (TIFF) [file pntd.0013487.s015.tiff]

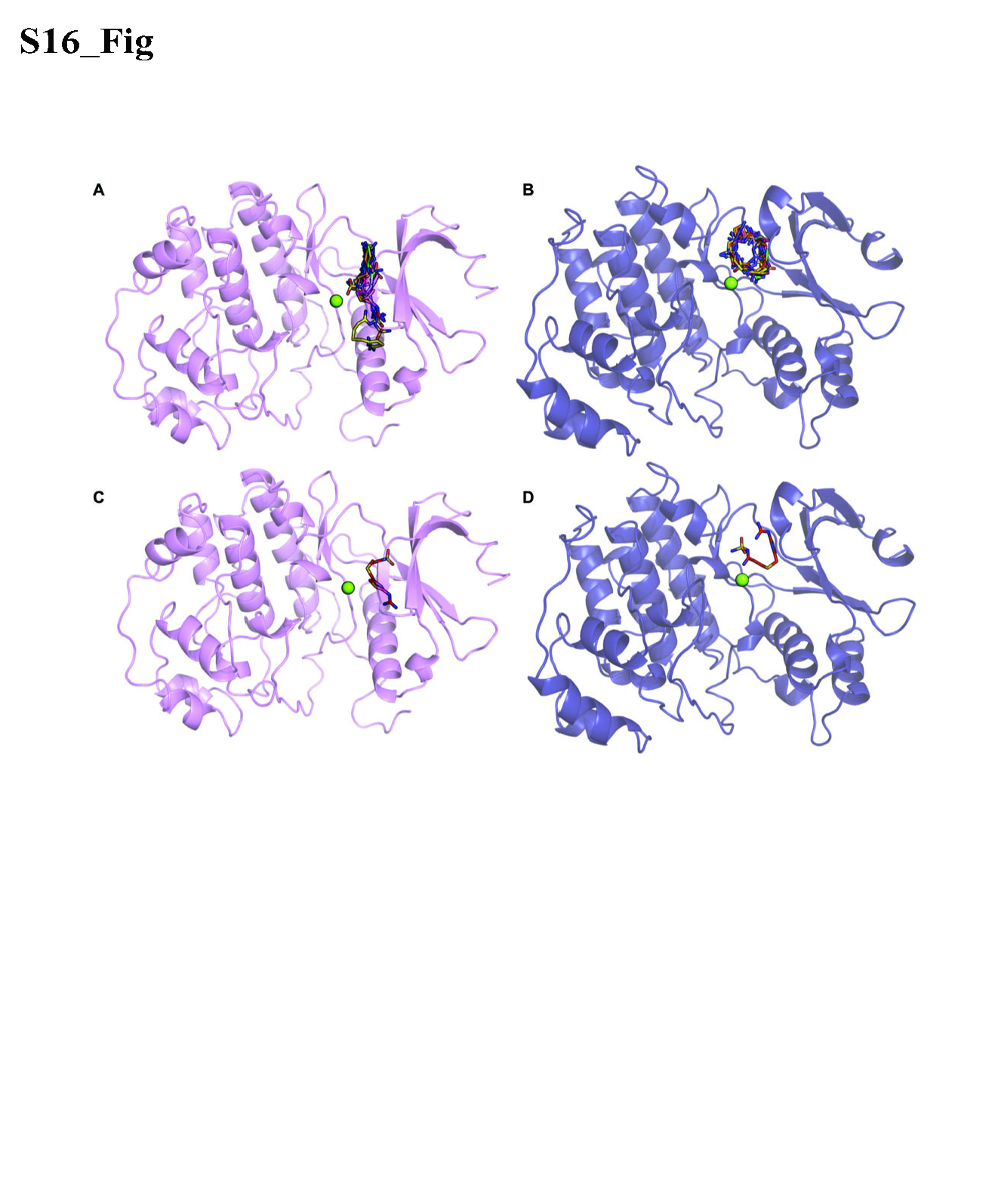

Supplement: S16 Fig — (A) TbERK8 docking results with all poses displayed as stick structures and colored by energy scores as in S10 Fig, with the Mg2+ ion as a green sphere. (B) HsERK8 docking results with all poses generated are displayed as stick structures and colored by energy scores, as in S10 Fig. HsERK8 is shown in navy cartoon with the Mg2+ ion as a green sphere. (C) TbERK8 docking results with best pose (#1) displayed as stick structures with red carbons, with the Mg2+ ion as a green sphere. (D) HsERK8 docking results with best pose (#1) displayed as stick structures with carbons colored in red and the Mg2+ ion as a green sphere. (TIFF) [file pntd.0013487.s016.tiff]

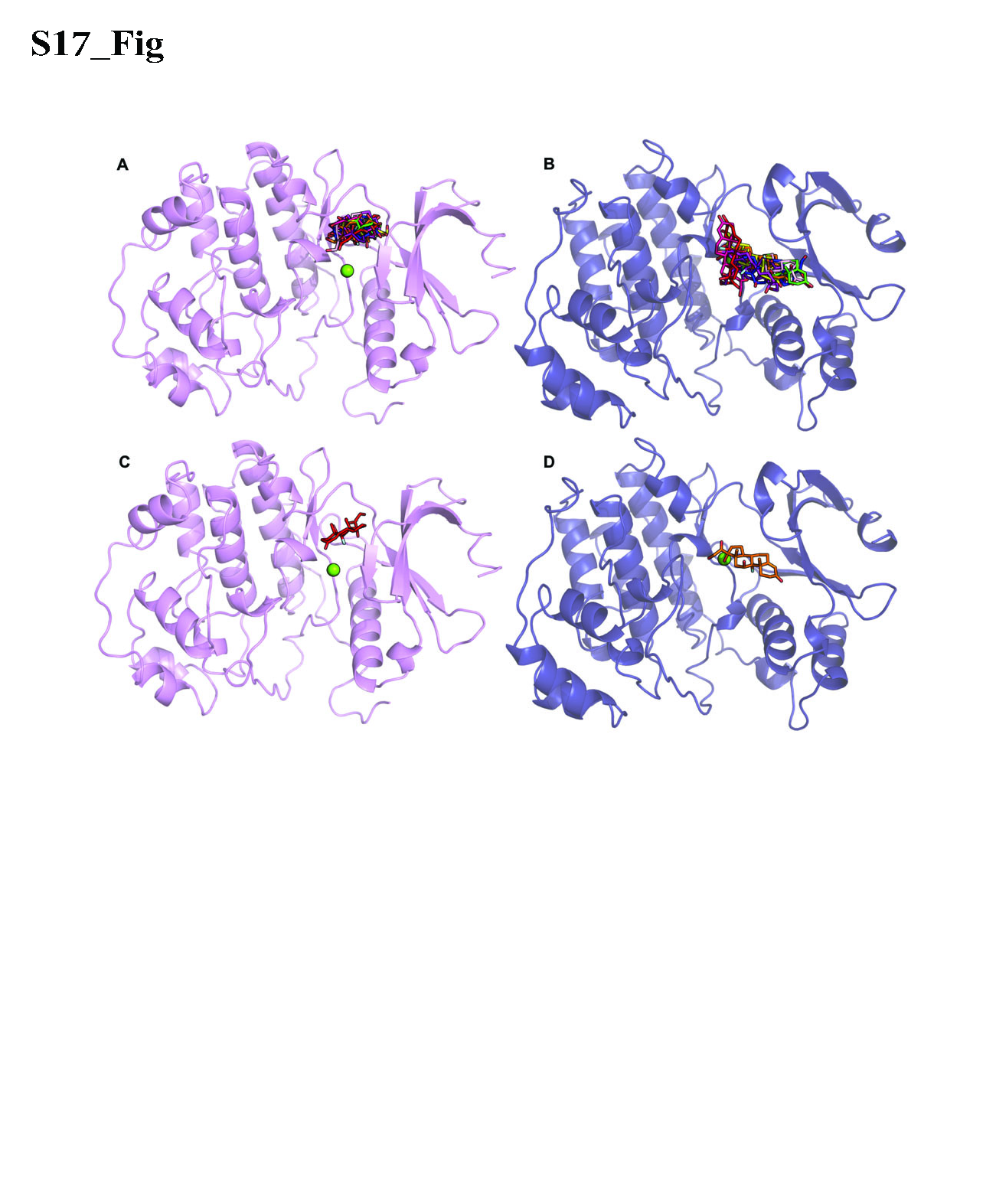

Supplement: S17 Fig — (A) TbERK8 docking results with all poses generated are displayed as stick structures and colored by energy scores, as in S10 Fig. (B) HsERK8 docking results with all poses displayed as stick structures and colored as in S10 Fig. (C) TbERK8 docking results with best pose (#1) displayed as stick structures and colored as in S10 Fig. HsERK8 is shown in navy cartoon with Mg2+ as a green sphere. (D) HsERK8 docking results with best pose (#2) displayed as stick structures and carbons colored in orange and by atom type with the Mg2+ ion as a green sphere. (TIFF) [file pntd.0013487.s017.tiff]

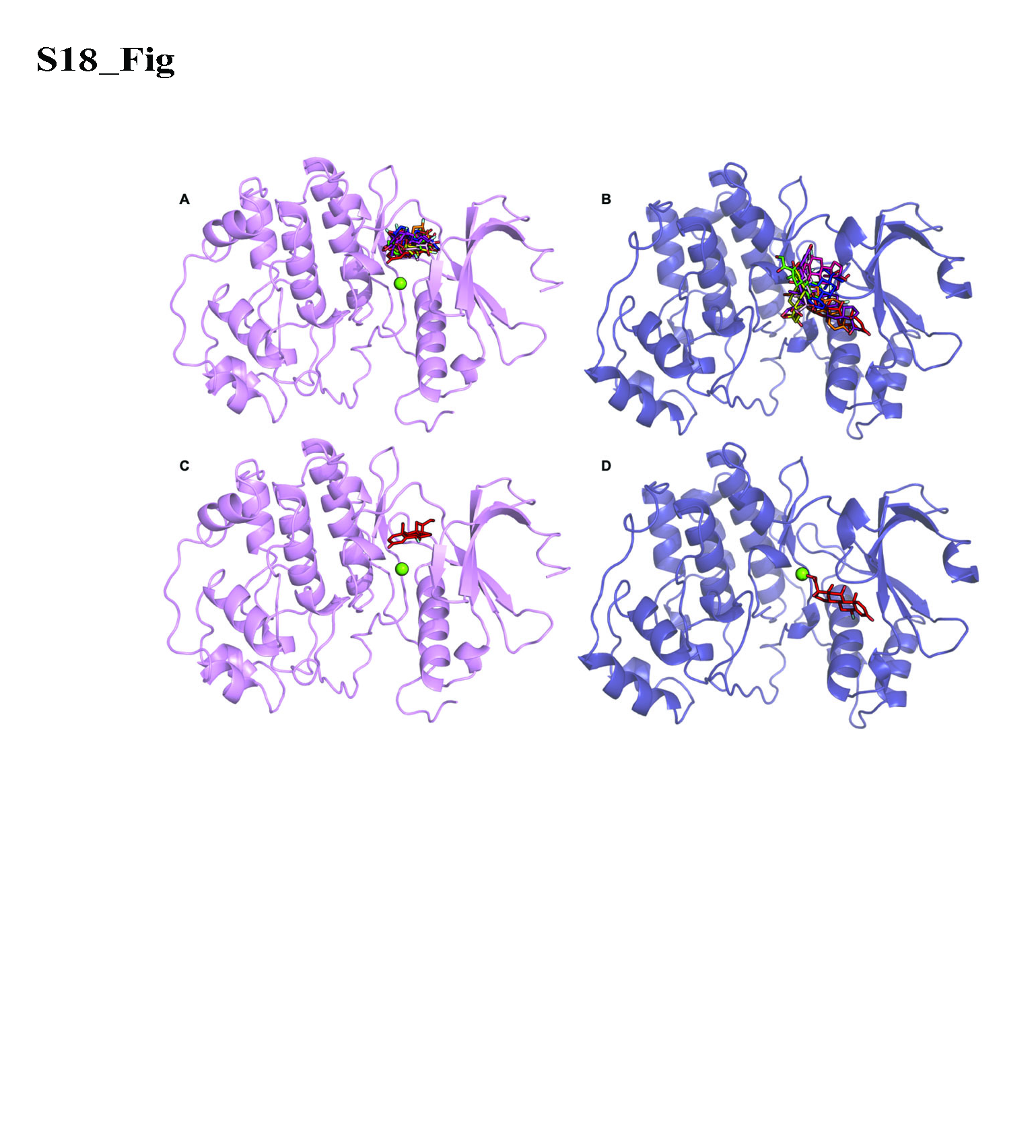

Supplement: S18 Fig — (A) TbERK8 docking results with all poses generated are displayed as stick structures and colored by energy scores as in S10 Fig. (B) HsERK8 docking results with all poses generated are displayed as stick structures and colored by energy scores, as in S10 Fig. (C) TbERK8 docking results with the best pose (#1) are displayed as stick structures, as in S10 Fig. HsERK8 is shown in navy cartoon in S10 Fig. (D) HsERK8 docking results with best pose (#1) displayed as stick structures and colored as in S10 Fig. (TIFF) [file pntd.0013487.s018.tiff]

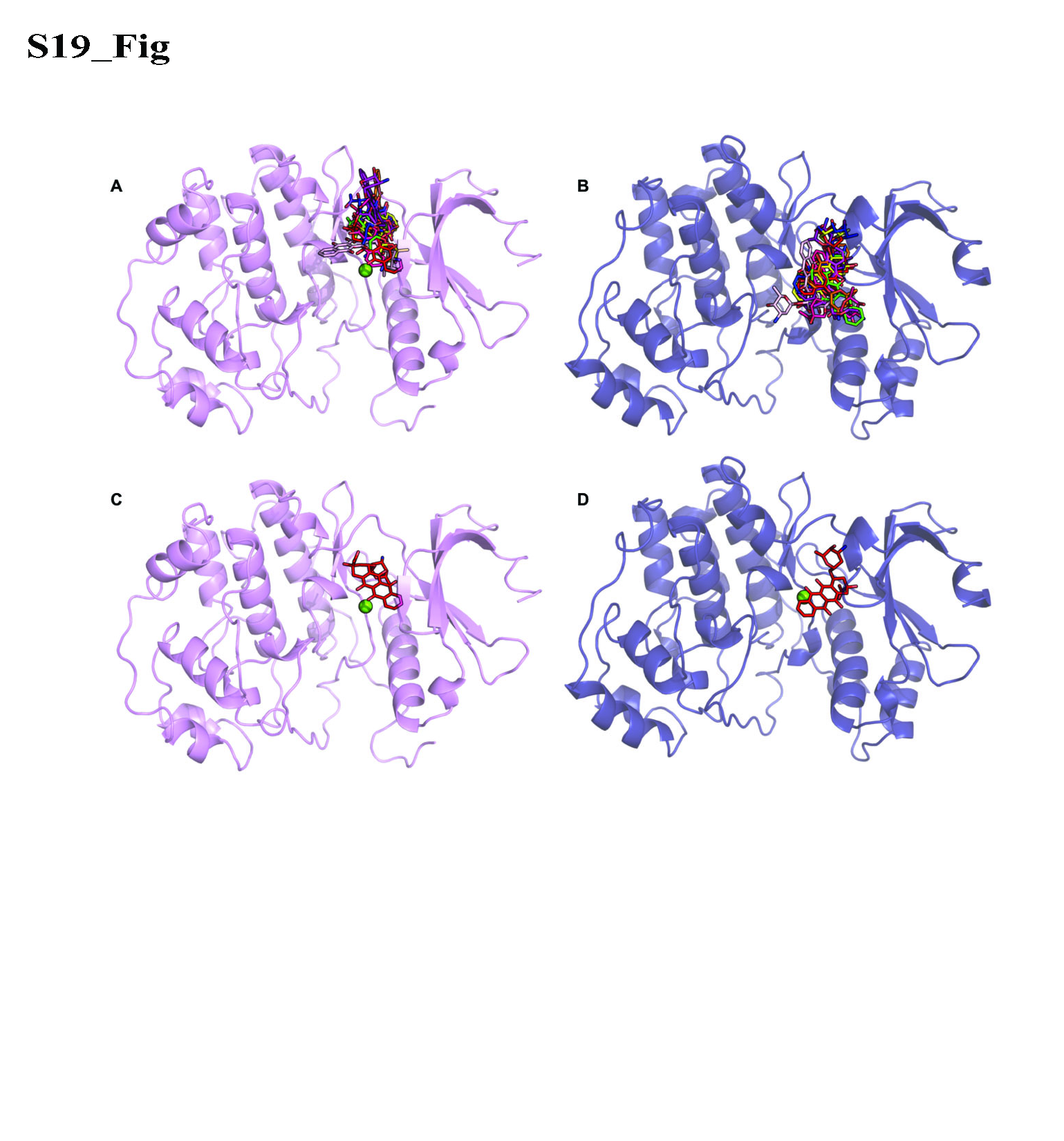

Supplement: S19 Fig — (A) TbERK8 docking results with all poses generated are displayed as stick structures and colored as in S10 Fig. (B) HsERK8 docking results with all poses generated are displayed as stick structures and colored as in S10 Fig. (C) TbERK8 docking results with best pose (#1) displayed by its stick structure and colored as in S10 Fig. HsERK8 is shown as navy and colored, as shown in S10 Fig. (D) HsERK8 docking results with best pose (#1) displayed by its stick structure and colored as in S10 Fig. (TIFF) [file pntd.0013487.s019.tiff]

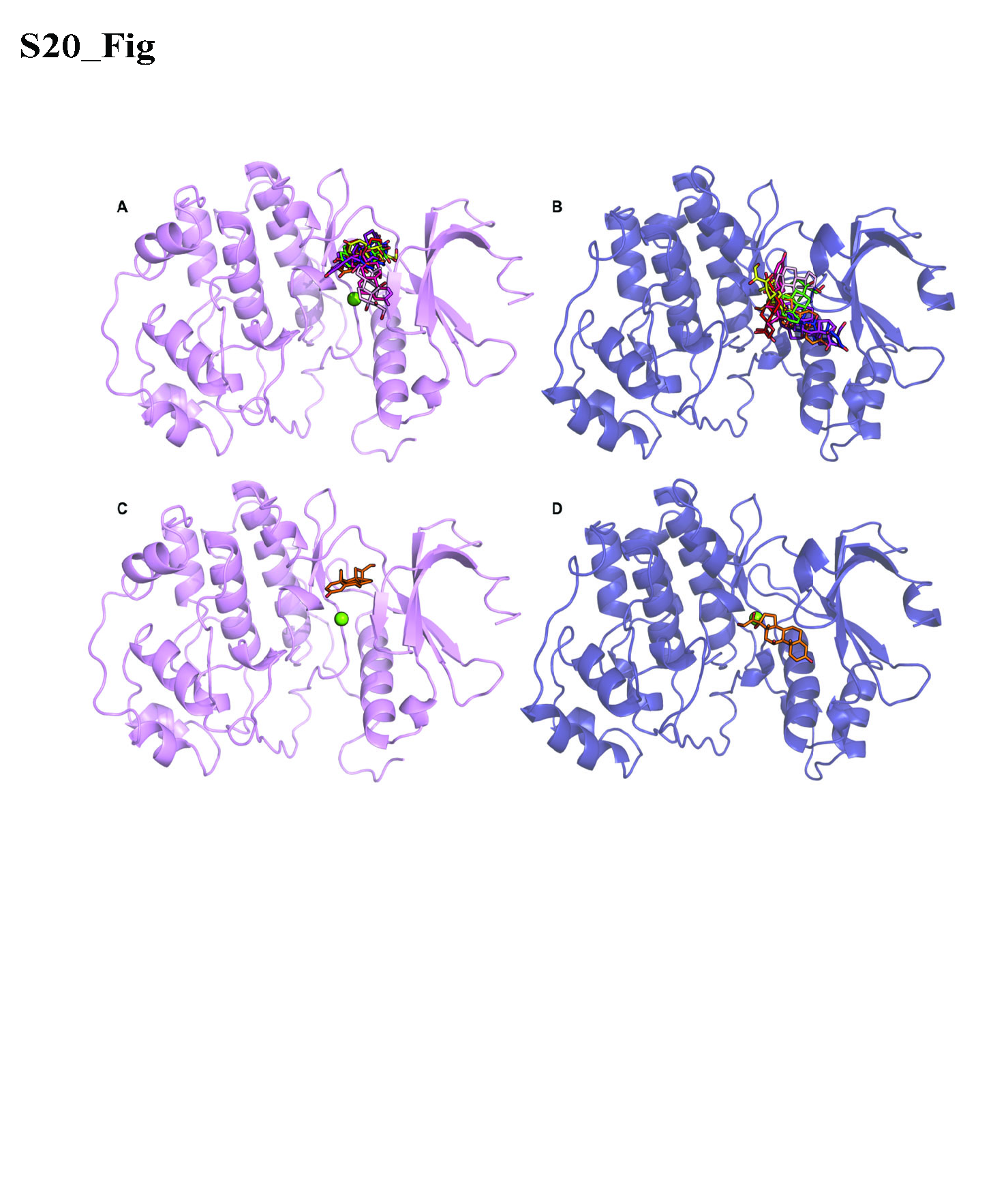

Supplement: S20 Fig — (A) TbERK8 docking results with all poses displayed as stick structures and colored as in S10 Fig. (B) HsERK8 docking results with all poses displayed as stick structures and colored as in S10 Fig. HsERK8 is shown as a navy cartoon and colored as in S10 Fig. (C) TbERK8 docking results with best pose (#2) displayed by its stick structure and colored as in S10 Fig. (D) HsERK8 docking results with the best pose (#2) displayed by its stick structure and colored as in S10 Fig. (TIFF) [file pntd.0013487.s020.tiff]

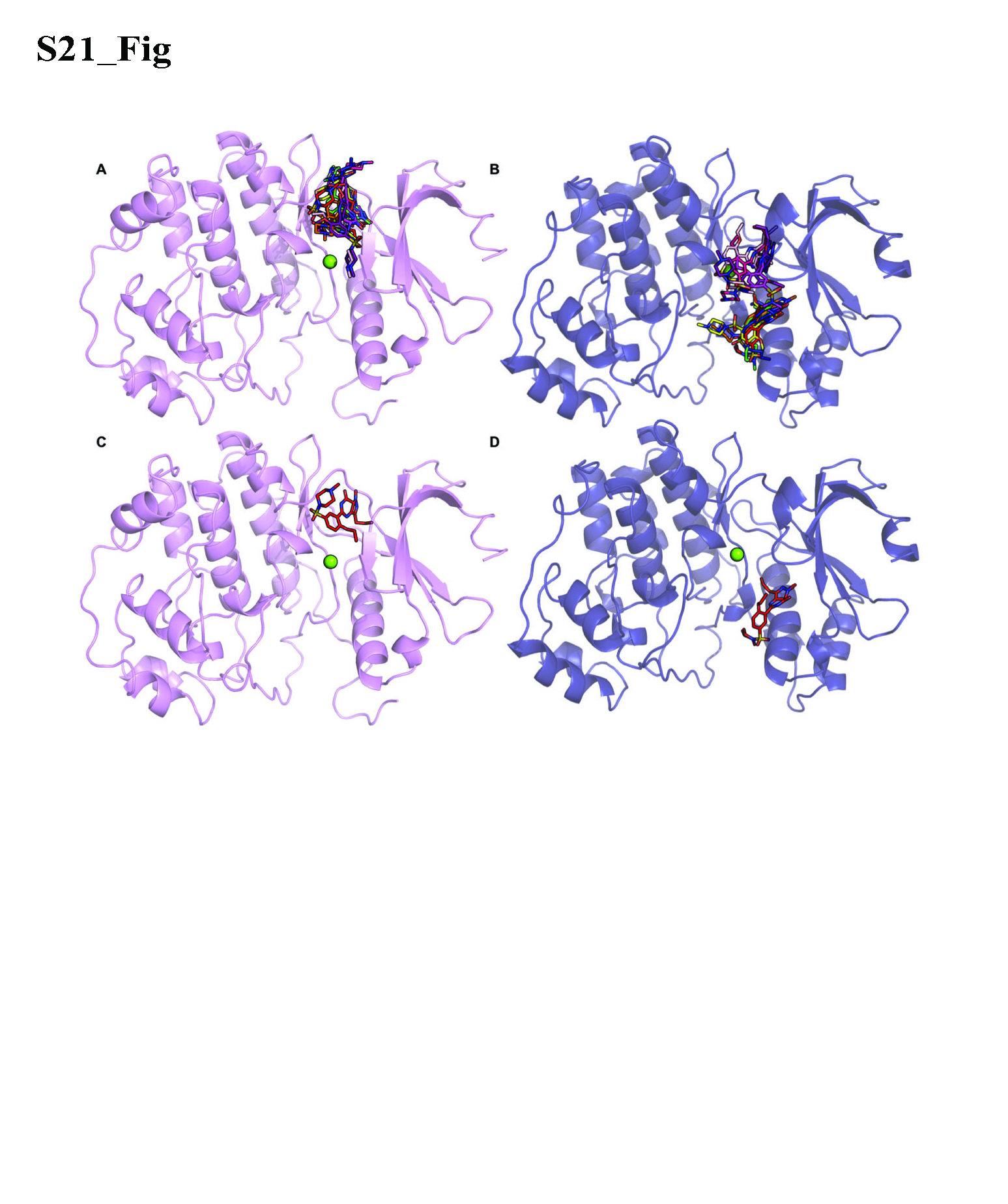

Supplement: S21 Fig — (A) TbERK8 docking results with all poses displayed as stick structures and colored as in S10 Fig. (B) HsERK8 docking results with all poses displayed as stick structures and colored as in S10 Fig. HsERK8 is shown as a navy cartoon and colored as in S10 Fig. (C) TbERK8 docking results with the best pose (#1) displayed by its stick structure and colored as in S10 Fig. (D) HsERK8 docking results with the best pose (#1) displayed by its stick structure and colored as in S10 Fig. (TIFF) [file pntd.0013487.s021.tiff]

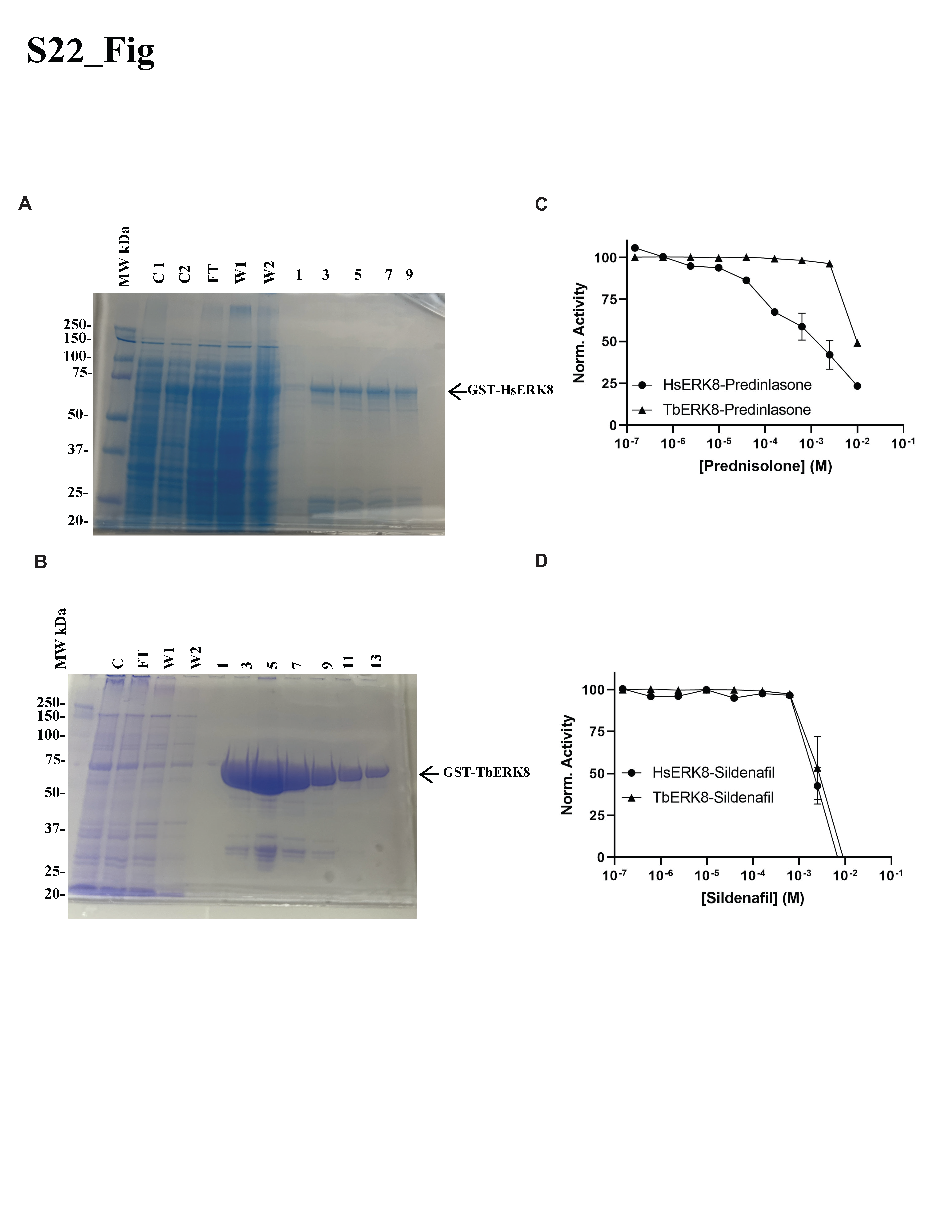

Supplement: S22 Fig — (A) Purification of GST-HsERK8 by GST-agarose column chromatography after overexpression in E. coli. Arrow points to eluted fractions containing ~84 kDa GST-HsERK8 fusion protein stained in SDSPAGE with Bio-Rad QC Colloidal Coomassie G-250. (B) Purification of GST-Tb ERK8 by GST-agarose column chromatography after overexpression in E. coli. Arrow points to eluted fractions containing ~74 kDa GST-TbERK8 fusion protein stained in SDSPAGE with Coomassie Brilliant Blue R-250 (For SDSPAGE, crude lysate [C], flow through [FT], wash [W], numbered lanes are eluted fractions). (C) Dose curves for kinase assays comparing prednisolone inhibition of HsERK8 and TbERK8. (D) Dose curves for kinase assays comparing Sildenafil inhibition of HsERK8 and TbERK8. Curves were generated by Prism 8.1.1 (GraphPad) from kinase assays testing 4-fold serial dilution of each drug at concentrations ranging from 10 mM to 150 nM for their inhibitor potency. Each point in the dose curve represents the mean value with standard deviation from three independent assays done on different days. (TIFF) [file pntd.0013487.s022.tiff]

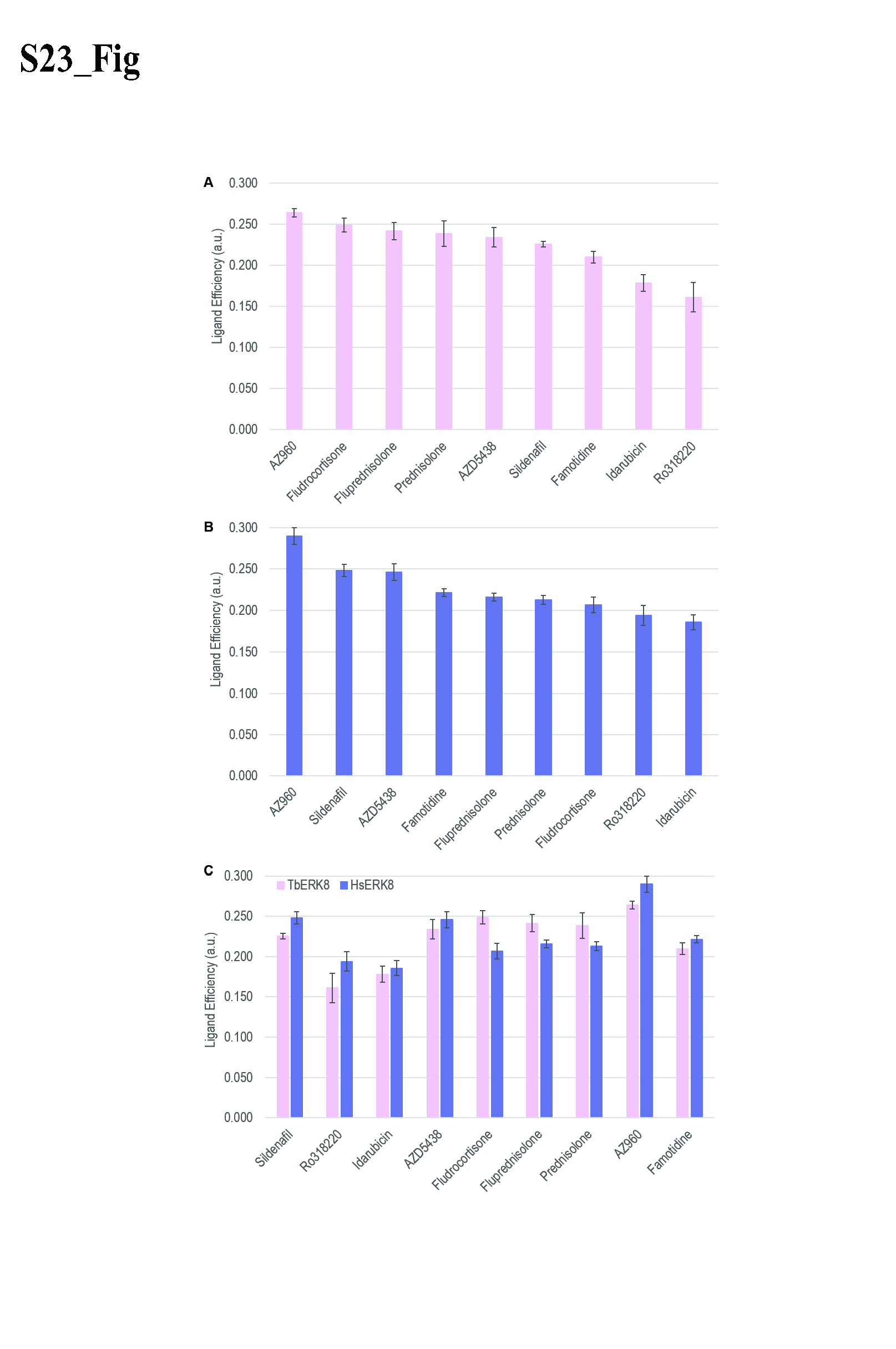

Supplement: S23 Fig — Ligand efficiencies are calculated in kcal/mol/heavy atom count. (A) TbERK8 ligand efficiencies are organized from highest efficiency to lowest. (B) HsERK8 ligand efficiencies are organized from highest efficiency to lowest. (C) Comparison of TbERK8 and HsERK8 ligand efficiencies organized by size (Å3) of each ligand. Ligand efficiency calculated using all poses output energies from AutoDock Vina and heavy atom numbers and standard deviations. (TIFF) [file pntd.0013487.s023.tiff]

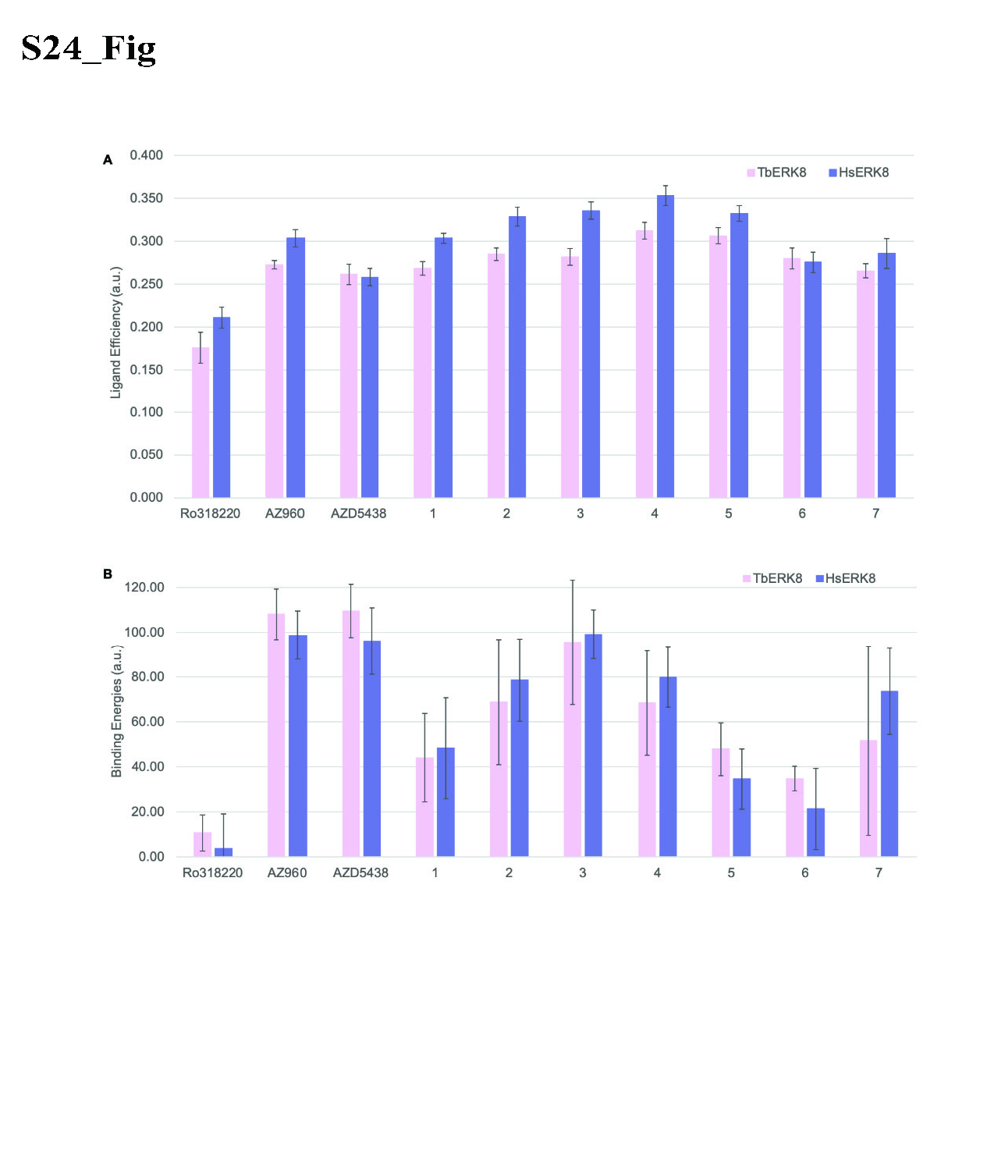

Supplement: S24 Fig — (A) Ligand efficiency was calculated using all pose output energies from AutoDock Vina. (B) Binding energies were evaluated using all pose outputs from AutoDock Vina and Schrödinger-Maestro. The molecular mechanics/generalized Born surface area (MM-GBSA) feature was used to calculate the energy of complexes and standard deviations. (TIFF) [file pntd.0013487.s024.tiff]

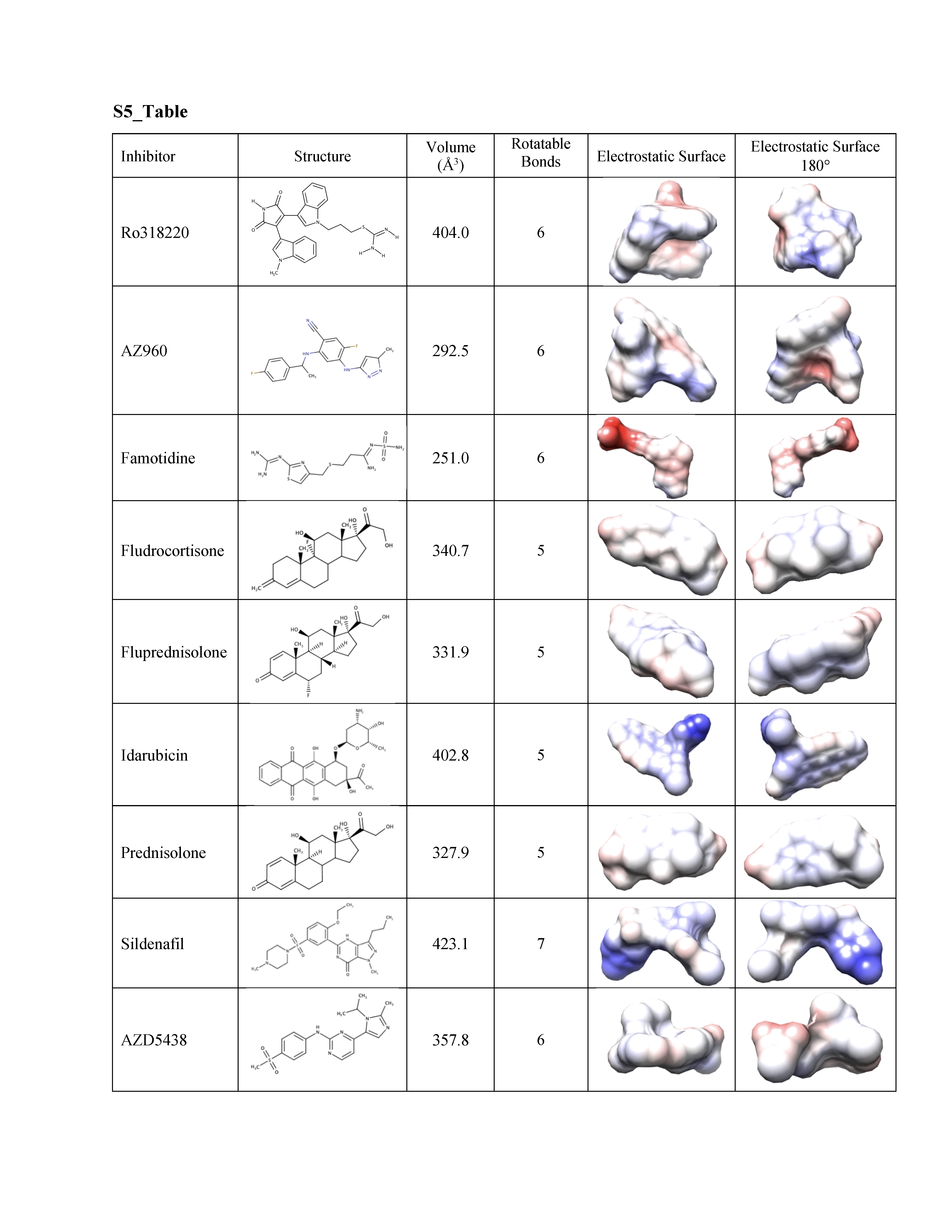

Supplement: S5 Table — (TIFF) [file pntd.0013487.s030.tiff]

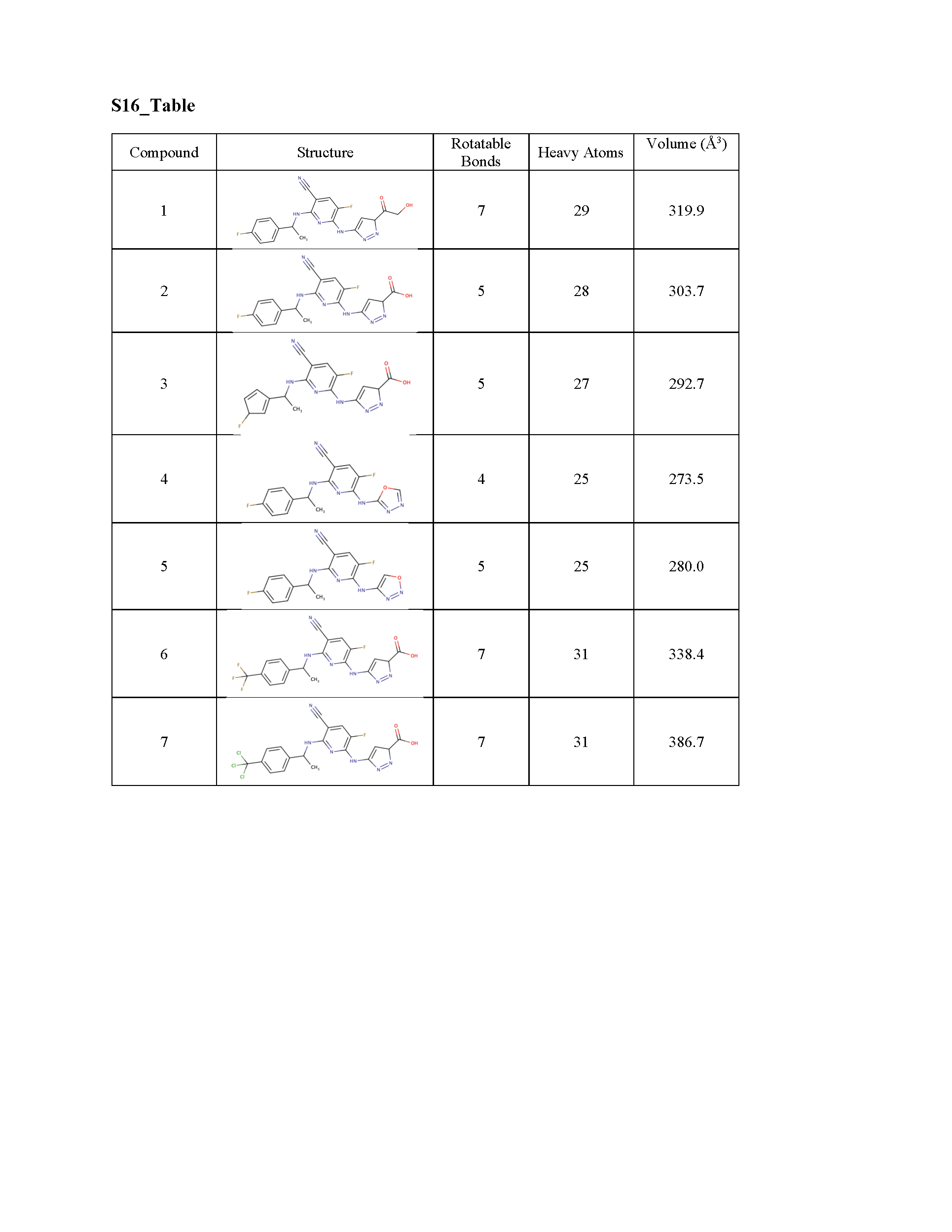

Supplement: S16 Table — (TIFF) [file pntd.0013487.s041.tiff]
