# Supplementary material for: Molecular docking to homology models of human and Trypanosoma brucei ERK8 that identified ortholog-specific inhibitors
Source: PLoS Negl Trop Dis. 2025 Sep 12;19(9):e0013487. doi: 10.1371/journal.pntd.0013487 (PMC12445744; doi:10.1371/journal.pntd.0013487)
Supplement: S1 File — (DOCX) [file pntd.0013487.s045.docx]

**Supporting Experimental Methods**

**Culturing of *T. brucei***

Bloodstream form parasites (90-13 strains(1) were incubated in 5% CO_2_ at 37 °C in HMI-9 medium modified to contain 20% fetal bovine serum(2). The parasites were cultured in media containing 100 U/ml penicillin/ and 100 μg/ml streptomycin. The selection medium contained 5 μg/ml of hygromycin B, 2.5 μg/ml of G418, and 2.5 μg/ml of phleomycin. The recombinant open reading frames were subcloned into the Hind III and AflII restriction sites of the C-terminal HA-tagged modified version of pLEW111(3). Recombinant proteins were overproduced in *T. brucei* 90-13 strains in HMI-9 medium containing 100 ng/ml of tetracycline, incubated in humidified chambers at 37 °C with 5% CO_2_ for 24 hours.

**Immunoblots**

Parasites were lysed with Tb lysis buffer (1.0% Triton X-100, 10 mM Tris pH 7.5, 25 mM KCl, 150 mM NaCl, 1 mM MgCl_2_, 0.2 mM EDTA, 1 mM dithiothreitol, 20% glycerol). Twenty-five micrograms of crude lysate were resolved by SDS-PAGE and transferred to a polyvinylidene difluoride (PVDF) membrane. After transferring and blocking, the PVDF membrane was incubated with rabbit anti-HA (1:2,000 dilution) for 1 h and washed three times for 5 min each with TBST (10 mM Tris, pH 7.4, 150 mM NaCl, 0.4% Tween 20). After the third wash, horseradish peroxidase-conjugated donkey anti-rabbit IgG (diluted 1:1,000) was added to the blots for 1 h. The blots were then rewashed in the same buffer 3x for 5 min each and examined using Pierce ECL chemiluminescence (Cat # 32209 Life Technologies, Carlsbad, CA).

**TbERK8 mutant constructs**

Wild-type TbERK8 was amplified with the forward primer 5’-CGCGCCAAGCTTATGTCATCAGAAATAGAGCC-3' and the reverse primer 5’-CTTAAGTTTGTGCAACACACGAGAGGC-3'. Point mutations were made in TbERK8 cDNA by the annealing overlapping PCR method(4). PCR templates used for the annealing overlapping reaction were made by combining the wild-type TbERK8 forward primer with the appropriate mutant reverse primer or the wild-type TbERK8 reverse primer with the appropriate mutant forward primer listed below:

K42A rev 5’-GCGTCGTATATCTTCGCTAACGCTACAACC-3’

K42A fwd 5’-GGTTGTAGCGTTAGCGAAGATATACGACGC-3’

D155A rev 5’-TTGAGCAATCGCTGTTTACAAGCA-3’

D155A fwd 5’-TGAAAGTGGCAGCCTTTGGGCTAG-3’. The full-length mutant TbERK8 cDNAs were subcloned into the vector's AlfII and HindIII sites and expressed in pLEW1113HA.

**Kinase immuno-precipitation assay**

Extracts from at least 5x10^7^ induced parasites were pelleted by centrifugation and lysed with 1 ml of immune-precipitation (IP) lysis buffer on ice for 30 min (1% Triton X-100, 10 mM Tris pH 7.9, 25 mM KCl, 150 mM NaCl, 1 mM MgCl_2_, 0.2 mM EDTA, 1 mM dithiothreitol (DTT), 20% glycerol and 1 complete protease inhibitor tablet (Cat # 11836170001 Sigma-Aldrich, Inc. St. Louis, Mo.). Lysates were precleared with 50 μl of Protein A beads. Precleared lysates were transferred to new tubes, and 5 μl of mouse anti-HA antiserum (Cat # H9658, Sigma-Aldrich, Inc., St. Louis, MO) was added to the mixture and incubated for 5 h. 50 μl of a 50% protein-A agarose bead slurry was added to the mixture and rotated overnight at 4 °C. The beads were washed 5 times with 1 mL of IP lysis buffer. After the last wash, beads were resuspended in 50 μl of IP lysis buffer. Ten microliters were taken from the slurry to perform a kinase IP reaction using Buffer B (30 mM Tris, 10 mM MgCl_2_, 1 mM DTT, 5% glycerol, and 0.1 mg/ml BSA) with 10 μCi of 32P-γ-ATP in 30 μl reactions. Reactions were stopped by adding 5X SDS-PAGE loading buffer and boiling for 2-5 minutes. Ten microliters of the kinase IP reaction were resolved by SDS-PAGE and examined by autoradiography. Quantification was conducted using a Storm 820 PhosphorImager (GE Life Sciences). For kinase assays using recombinant TbERK8, 100 ng of enzyme was used in 30 μl reactions using identical conditions as kinase IP reactions.

**Introduction of transgenes into *T. brucei***

Expression of TbERK8_HA_ constructs in the bloodstream form of *T. brucei*. The plasmid pLEW11TbERK8_3HA_ described in Valenciano et al.(5) allows for tetracycline-inducible overexpression of hemagglutinin A-tagged TbERK8_HA_ in *T. brucei.* For electroporation, 10^7^ parasites were pelleted by centrifugation and washed once with 10 ml of phosphate-buffered saline (4.3 mM Na_2_HPO_4,_ 137 mM NaCl, 2.7 mM KCl, 1.4 mM KH_2_PO_4_), pH 7.4. 1-to-10 μg of Not I linearized plasmid was nucleofected into *T. brucei* using Lonza Nucleofector II and the T-cell kit (Cat # VPA-1002, Greenwood, SC). The parasites were pulsed using program X-001, then transferred to 10 ml of modified HMI-9 or Cunningham's media and incubated overnight at 37 °C with 5% CO_2_. The next day, stable clones were selected by limiting dilutions in 24-well tissue culture plates in media containing 5.0 μg/ml hygromycin B, 2.5 μg/ml G418, and 2.5 μg/ml of phleomycin.

**Reference**

1. Wirtz, E., Leal, S., Ochatt, C., andCross, G. A. (1999) A tightly regulated inducible expression system for conditional gene knock-outs and dominant-negative genetics in Trypanosoma brucei Mol Biochem Parasitol **99**, 89-101, <http://www.ncbi.nlm.nih.gov/pubmed/10215027>

2. Hirumi, H., andHirumi, K. (1989) Continuous cultivation of Trypanosoma brucei blood stream forms in a medium containing a low concentration of serum protein without feeder cell layers The Journal of parasitology **75**, 985-989, <http://www.ncbi.nlm.nih.gov/pubmed/2614608>

3. Motyka, S. A., Drew, M. E., Yildirir, G., andEnglund, P. T. (2006) Overexpression of a cytochrome b5 reductase-like protein causes kinetoplast DNA loss in Trypanosoma brucei J Biol Chem **281**, 18499-18506 10.1074/jbc.M602880200

4. Bryksin, A. V., andMatsumura, I. (2010) Overlap extension PCR cloning: a simple and reliable way to create recombinant plasmids Biotechniques **48**, 463-465 10.2144/000113418

5. Valenciano, A. L., Knudsen, G. M., andMackey, Z. B. (2016) Extracellular-signal regulated kinase 8 of Trypanosoma brucei uniquely phosphorylates its proliferating cell nuclear antigen homolog and reveals exploitable properties Cell Cycle **15**, 2827-2841 10.1080/15384101.2016.1222340
